# Impact of screening programmes for type 1 diabetes in youth: A systematic review and meta‐analysis

**DOI:** 10.1111/dme.70236

**Published:** 2026-01-31

**Authors:** Roberto Franceschi, Enza Mozzillo, Marco Marigliano, Giulio Maltoni, Ernesto Maddaloni, Rocco Amendolara, Luca Bernardini, Riccardo Bonfanti, Federica Ciambrelli, Francesca Di Candia, Ludovica Fedi, Alessandro Fierro, Dario Iafusco, Antonio Iannilli, Letizia Leonardi, Claudio Maffeis, Evelina Maines, Riccardo Pertile, Claudia Piona, Ivana Rabbone, Carlo Ripoli, Andrea Scozzarella, Valentina Tiberi, Raffaella Buzzetti, Valentino Cherubini

**Affiliations:** ^1^ CISMed ‐ Centro Interdipartimentale di Scienze Mediche University of Trento Trento Italy; ^2^ Department of Translational Medical Science University of Naples Federico II Naples Italy; ^3^ Department of Surgery, Dentistry, Pediatrics and Gynecology University and Azienda Ospedaliera Universitaria Integrata of Verona Verona Italy; ^4^ Pediatric Unit IRCCS Azienda Ospedaliero‐Universitaria di Bologna Bologna Italy; ^5^ Department of Experimental Medicine Sapienza University Rome Italy; ^6^ Department of Medical and Surgical Sciences DIMEC University of Bologna UNIBO Bologna Italy; ^7^ Department of Pediatrics Diabetes Research Institute, IRCCS San Raffaele Milan Italy; ^8^ Department of Pediatrics Università Vita Salute San Raffaele Milan Italy; ^9^ Department of Woman, Child and General and Specialistic Surgery, Regional Center of Pediatric Diabetes University of Campania “L. Vanvitelli” Naples Italy; ^10^ Department of Women's and Children's Health Azienda Ospedaliero‐Universitaria delle Marche, “G. Salesi Hospital” Ancona Italy; ^11^ Department of Clinical and Evaluative Epidemiology APSS Trento Italy; ^12^ Department of Health Sciences University of Piemonte Orientale Novara Italy; ^13^ Pediatric Diabetology Unit, Department of Pediatrics ASL 8 Cagliari Cagliari Italy

**Keywords:** anti‐islet antibody, DKA risk, meta‐analysis, screening, systematic review, type 1 diabetes

## Abstract

**Aim:**

To evaluate the impact of anti‐islet antibody (IAb) screening on the general population and first‐degree relatives (FDRs)/high‐risk individuals and evidence‐based follow‐up modalities.

**Methods:**

We performed this review through systematic searches of PubMed, EMBASE, Cochrane Library, Web of Science, ClinicalTrials.gov and International Clinical Trials Registry Platform between 15 March 2006, and 15 March 2025. We selected studies on children and adolescents screened for T1D IAbs, compared with people who were not screened or IAb+ individuals who were not followed up. PICOS framework was used in the selection process. Outcome data were extracted, and a meta‐analysis of DKA risk at T1D onset was performed. Quality of evidence was assessed using the GRADE approach. This study was registered with PROSPERO, CRD42024523781.

**Results:**

Sixty‐six studies, 53 of moderate‐to‐high quality, were included. Screening was associated with lower DKA rates by 23% (95% CI 18–29%, *I* ^2^ = 88.8%). The risk of stage 3 T1D progression was high in younger children with persistent and/or multiple IAb+. Screening was associated with higher indicators of parental anxiety, which decreased during follow‐up. Children with IAb positivity were monitored according to age and T1D stage, using HbA1c, oral glucose tolerance testing and continuous glucose monitoring (CGM). Time above 140 mg/dL was a biomarker of progression.

**Conclusions:**

Population screening with IAbs and follow‐up of IAb+ individuals helps decrease DKA and allows participation in intervention trials. This systematic review provides evidence for clinical practice on the screening timing, modalities and follow‐up. Further studies on the use of CGM are expected.

**What's new**

- In this systematic review, 53 moderate‐high quality studies were included and revealed that screening for IAb reduces DKA by 23% (95% CI 18–29%).
- The risk of progressing to stage 3 T1D is particularly high in children aged 2–5 years with persistent IAb+ and/or multiple IAb+.
- Screening could increase parental anxiety, but this decreases after 1 year or during follow‐up.

## 1 INTRODUCTION

Type 1 diabetes (T1D) is the most common form of diabetes in children and adolescents.^1^ Screening for T1D in at‐risk children and the general population is becoming increasingly common worldwide due to the importance of identifying and treating at‐risk children during the initial stages of the disease.^2^, ^3^ Individuals considered at risk include first‐degree relatives (FDRs) of children and adolescents with T1D (as parents, siblings, children), or those with HLA genetic risk.

Studies of individuals at risk for developing T1D have demonstrated that the disease is a continuum that progresses sequentially through distinct identifiable stages before the onset of symptoms. Stage 1 is defined as the presence of β‐cell autoimmunity as evidenced by the presence of two or more islet autoantibodies (IAb+) with normoglycaemia, Stage 2 as the presence of β‐cell autoimmunity with dysglycaemia and Stage 3 as the onset of symptomatic disease.^4^

Screening children in the preclinical phase of T1D for the presence of IAb+ could help to identify at‐risk individuals and to follow them up, thereby preventing diabetic ketoacidosis (DKA), which still occurs in 15 to 70% of children at diabetes onset, incurring considerable morbidity, mortality and need for hospitalization.^5^ An early diagnosis, Stage 1 or Stage 2 T1D, provides the opportunity to educate children and their families about the signs and symptoms of Stage 3 T1D and/or to participate in clinical trials to prevent or delay progression to Stage 3 T1D.^3^

There are still several challenges to achieving nationwide screening, including establishing optimal timings, IAb testing, follow‐up modalities, costs and psychological issues. While there are consensus guidelines for monitoring individuals testing positive for IAb+^6^, ^7^, ^8^ and narrative reviews about the impact of T1D screening in the paediatric population, a systematic review of the topic is lacking.^9^, ^10^

To address this knowledge gap, we conducted a systematic review with the primary outcome being the impact of IAb screening for T1D in the paediatric population on the frequency of IAb+, T1D, DKA at T1D onset and psychological outcomes; secondary outcomes were timings of screening and follow‐up, modality of follow‐up, metabolic monitoring and prediction of T1D onset.

## 2 METHODS

The protocol of this systematic review was registered in PROSPERO on 23 March 2024, with accession CRD42024523781.

### 2.1 Search strategy

The PubMed, EMBASE, Cochrane Library, Web of Science, ClinicalTrials.gov and International Clinical Trials Registry Platform databases were searched on April 30, 2025, for studies published between 15 March 2006 and 15 March 2025, using the MeSH terms reported in the Supplemental Methods.

### 2.2 Criteria for study selection, outcomes and measures of effect

The literature was systematically searched according to the Population, Intervention, Comparison, Outcomes, Study design (PICOS) model (Table 1).

**TABLE 1:** PICOS strategy for the literature searches.

Population	Children and adolescents in the general population (birth to 18 years) Child and adolescents first‐degree relatives (FDR) of individuals with T1D Children and adolescents with a genetic risk of T1D
Intervention	Screening for T1D using multiple anti‐islet antibodies (IAb): anti‐insulin (IAA), glutamate decarboxylase (GAD), islet antigen 2 (IA‐2), and islet‐specific zinc transporter (ZnT8)
Comparison	Population not screened/not exposed to the intervention; standard care did not include follow‐up protocols for youths who tested positive during screening
Outcomes	Primary outcome: impact of screening on early T1D diagnosis and presentation Secondary outcomes: follow‐up modality; anxiety A description of the measures of effect for each outcome are reported in the text
Study design	RCTs, observational studies, case series (reviews excluded after the screening of references) Language: languages other than English are not an ‘a priori’ exclusion criterion Published after 2006

The primary outcome was screening effect on Stage 3 T1D diagnosis and presentation, and the measures of effect were (i) frequencies of IAb+ in the population screened and Stage 1–3 T1D; (ii) HbA1c, glucose and C‐peptide values at diagnosis; (iii) frequency of DKA, morbidity and mortality; and (iv) psychological outcomes (in parents and youth) such as family distress and burden, anxiety and depression.

Secondary outcomes were follow‐up modality, and the measures of effect were (i) age; (ii) timing of follow‐up; (iii) modality assessed by IAb and metabolic monitoring (oral glucose tolerance testing (OGTT), C‐peptide, HbA1c, continuous glucose monitoring (CGM), random serum blood glucose (BG)). Inclusion and exclusion criteria and the characteristics evaluated in each included study are reported in the Supplemental Methods.

### 2.3 Risk of bias (quality) assessment

We used the GRADE approach to rank the quality of included evidence (www.gradeworkinggroup.org), as reported in the Supplemental Methods. For studies reporting more than one outcome, the quality of the evidence was ranked for each outcome.

### 2.4 Data synthesis strategy

**FIGURE 1:**
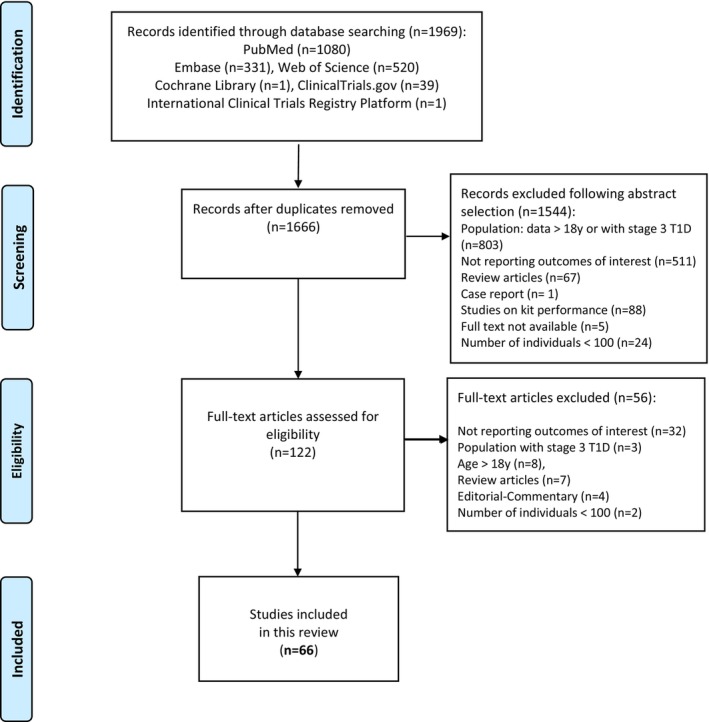
PRISMA flow diagram.

The PRISMA flow diagram summarizing the screening process is reported in Figure 1. The impact of the screening programme on the frequency of DKA at T1D onset was quantified through meta‐analysis (Supplemental Methods).

## 3 RESULTS

A literature review identified 1969 studies; after reviewing titles and abstracts, 122 full‐text manuscripts were assessed for eligibility (Figure 1). After full‐text examination, 56 studies were excluded (Table S1), leaving 66 studies for systematic review.

Of the 66 eligible studies, one was a randomized controlled trial (RCT), and there were 59 prospective longitudinal, four retrospective, and two cross‐sectional studies. Twenty‐three to 6193 individuals with IAb+ positivity were enrolled, with smaller sample sizes for CGM (range 23–105 individuals) and intravenous glucose tolerance testing (IVGTT; 47–685 individuals) studies. The summary and grading of the evidence are reported in Tables S2 and S3.

Ten papers reported data from studies enrolling the general population for screening: from health fairs,^11^ a cohort in Helsinki,^12^ Danish children,^13^ the Fr1da cohort in Bavaria‐Germany,^14^, ^15^, ^16^, ^17^ school children in northeast Germany,^18^ a Swedish cohort^19^ and the ASK study cohort.^20^ The other studies focused on paediatric individuals at high risk of T1D, that is, those with HLA‐susceptible genotypes and/or FDR with T1D.^21^, ^22^, ^23^, ^24^, ^25^, ^26^, ^27^, ^28^, ^29^, ^30^, ^31^, ^32^, ^33^, ^34^, ^35^, ^36^, ^37^, ^38^, ^39^, ^40^, ^41^, ^42^, ^43^, ^44^, ^45^, ^46^, ^47^, ^48^, ^49^, ^50^, ^51^, ^52^, ^53^, ^54^, ^55^, ^56^, ^57^, ^58^, ^59^, ^60^, ^61^, ^62^, ^63^, ^64^, ^65^, ^66^, ^67^

Fifty‐two studies were classified as moderate‐to‐high‐quality evidence. The main results of these studies, divided by outcome, are reported in Tables 2, 3, 4.

**TABLE 2:** Summary and grading of evidence of moderate‐to‐high‐quality studies for frequency of IAb+, T1D, and DKA and predictors, as detected by the screening programs.

References	Population	Age at screening, follow‐up duration	IAb+ (%)/reversion	T1D frequency and cumulative risk	DKA frequency	Level of evidence
*General population*						
Knip M et al. [12]	General population	3–18 y f/up: 27 y	1.0% (GADA+) 0.6% (IA‐2A+) 0.2% (GADA+ and IA‐2A+) Reversion: 0.4% for GADAs and 0.2% for IA‐2A over 6 y Inverse seroconversion (from positive to negative) rates were 33% and 57%, respectively	53% IAb+ developed Stage 3 T1D after a median pre‐dIAbetic period of 8.6 y Both GADA and IA‐2A positivity were associated with a relatively high probability (26% and 41%, respectively) of Stage 3 T1D after 27 y f/up	n.a.	Prevalence data and predictors ⊕ ⊕ ⊕ ⊖ moderate
Ziegler AG et al. [16]	General population	1.75–5.99 y f/up: 2.4 y	n.a.	0.31% had Stage 1 or Stage 2 T1D (of which 9.3% with stage 3) 3 y cumulative risk for Stage 3 T1D: 24.9%	3.2% of stage 3 T1D developed DKA	Prevalence data and predictors ⊕ ⊕ ⊕ ⊕ high
Hummel S et al. [14]	High risk (IAb+) vs. General population	4.6 y (median) f/up: 2.7 y	0.3% multiple IAb+ at the screening	27% of IAb+ developed Stage 3 T1D with a median of 2.3 y (1.1–4.3)	2.5% in the high‐risk population presented with DKA	Prevalence data ⊕ ⊕ ⊕ ⊖ moderate
Till AM et al. [18]	General population	6–17 y f/up of IAb+: 18 y	7.2% IAb+ at the screening At 1st f/up: ‐ 77% single IAb+ ‐ 23% multiple IAb+	17% for IAb+ children (15% with single IAb+, 85% with multiple IAb+) Cumulative risk for Stage 3 T1D at 10‐ and 18‐y f/up: 59.7% and 75.1% (multiple IAb+), 1.2% and 22.6% (single IAb+)	n.a.	Prevalence data and predictors ⊕ ⊕ ⊕ ⊕ high
Gullstrand C et al. [19]	General population	0–5 y f/up: 1, 2.5, 5 y of age	1.7% IAb+	0.18% of the GP developed Stage 3 T1D GADA+ or IA‐2A+ give PPV of Stage 3 T1D: 3 and 5%, respectively Both IAb+: give PPV of T1D of 11% at 5 y	n.a.	Prevalence data and predictors ⊕ ⊕ ⊕ ⊖ moderate
McQueen RB et al. [20]	General population	2–17 y f/up:3 y	0.48% multiple IAb 0.53% single IAb	n.a.	n.a.	Prevalence data ⊕ ⊕ ⊕ ⊖ moderate
*High risk/FDR*						
Bonifacio E et al. [21]	High risk/FDR	Landmark ages: 7.5 m 2.125 y 4.25 y 6.25 y 8.25 y f/up: up to 15 y of age	9.46% at median age of 3.2 y 5‐y risk: at 7.5 m: 6.3% for any IAb, 4.3% for multiple at 6.25 y: 3.2% for any IAb, 1.1% for multiple	3.7% Highest sensitivity and PPV of multiple IAb phenotypes for Stage 3 T1D was achieved by IAb screening at 2 y and again at 5–7 y of age	n.a.	Prevalence data and predictors ⊕ ⊕ ⊕ ⊕ high
Frohnert BI et al. [22]	High‐risk	≤ 2.5 y f/up: 15 y of age or T1D onset	5% developed >1 IAb at some point during f/up	62% of IAb+ progressed to Stage 3 T1D The 15‐y risk of progression to Stage 3 T1D varied from 18 to 88% based on the stringency of IAb+ definition, multiple IAbs, persistent IAbs, age at seroconversion	n.a.	Prevalence data ⊕ ⊕ ⊕⊖/⊕ ⊕ ⊕ ⊕ moderate/high Predictors ⊕ ⊕ ⊕ ⊕ high
Ghalwash M et al. [23]	High‐risk	10 y or 10 y and 14 y f/up: up to 18 y	23.4%	13.9% by the age 18 y Detection Stage 3 T1D by the age of 18 y: Single screening at 10 y: sens 90%, PPV 66% Double screening (10 and 14 y): sens 93%, PPV of 55%	n.a.	Prevalence data and predictors ⊕ ⊕ ⊕⊖/⊕ ⊕ ⊕ ⊕ moderate/high
Giannopoulou EZ et al. [24]	High risk/ FDR	0–20 y f/up 20 y (birth, 9 m and 2, 5, 8, 11, 14, 17 and 20 y)	8.9%	41% of IAb+ Stage 3 T1D 10‐y risk: IAA+ and developed multiple IAb later: 56% GADA+ and developed multiple IAb later: 53% multiple IAb 1st sample: 69%	n.a.	Prevalence data and predictors ⊕ ⊕ ⊕ ⊕ high
Helminen O et al. [25]	High‐risk IAb+	Since newborn f/up: 4.5–6 y before diagnosis	1162 (6.2% out of all included children) developed at least one IAb	> incidence of dysglycaemia in children progressing to Stage 3 T1D during f/up	n.a.	Prevalence data and predictors ⊕ ⊕ ⊕ ⊖ moderate
Hoffman VS et al. [26]	FDR	Landmark ages: at birth, 3.5, 6.5, 12.5 y f/up: 6 y	8%, 4.6%, 2.6% and 0.9%, at the landmark ages: birth, 3.5, 6.5 and 12.5 y, respectively	Risks of Stage 3 T1D within 6 y of f/up at these landmark ages were 5.3%, 2.9%, 1.8% and 1%, respectively	n.a.	Prevalence data and predictors ⊕ ⊕ ⊕ ⊕ high
Krischer JP et al. [27]	High‐risk	3 m‐15 y f/up: 15 y	6.5% until 6 y of age GADA only were marginally less likely to develop T1D in the first year after seroconversion vs. those who had IAA only (HR 0.35, 95% CI 0.12, 1.04, *p* = 0.06).	n.a.	n.a.	Prevalence data ⊕ ⊕ ⊕ ⊕ high Predictors ⊕ ⊕ ⊕ ⊖ moderate
Mahon JL et al. [28]	FDR	1–45 y f/up: 5 y	4.8% had ≥1 IAb+ in 1st sample	5.6% of individuals with ≥2 IAb+ in 1st sample or ≥1 Ab+ in 2nd sample	n.a.	Prevalence data ⊕ ⊕ ⊕⊖/⊕ ⊕ ⊕ ⊕ moderate/high
Ng K et al. [29]	High risk/ FDR	0–15 y f/up 15 y	6.5%	37.4% of IAb+ The 5‐y risk conferred by single and multiple IAbs could be stratified from 6 to 75% (*p* < 0.0001)	n.a.	Prevalence data and predictors ⊕ ⊕ ⊕ ⊖ moderate
Vehik K et al. [30]	FDR	1–17 y f/up: 5 y	5.5% at the initial screen. The cumulative seroconversion was 2% <10 y of age vs. 0.7% ≥10 y of age For each 1‐y, the risk of seroconversion decreased by 11% for IAA [*p* < 0.0001] and 4% for GAD65 [*p* = 0.04]	n.a.	n.a.	Prevalence data and predictors ⊕ ⊕ ⊕ ⊖ moderate
Vehik K et al. [31]	FDR	3–18 y f/up: 2.3 y (1.7–4.1)	4% in 2 y For each 1‐y increase in age in this cohort, the risk of any IAb seroconversion (HR 0.95, 95% CI 0.92–0.97) decreased by 5%, and for any two IAbs risk decreased by 13% (0.87, 0.82–0.93). The majority of children (75%) seroconverted by 13 y of age	n.a.	n.a.	Prevalence data ⊕ ⊕ ⊕⊖/⊕ ⊕ ⊕ ⊕ moderate/high Predictors ⊕ ⊕ ⊕ ⊖ moderate
Wentworth JM et al. [32]	High‐risk/ general population	15.7 ± 10.8 y f/up: –	Screened relatives: 652 (3.8%) had one IAb 306 (1.8%) had multiple IAb	178 (1%) had Stage 3 T1D	5% had DKA (HR) vs. 31%	Prevalence data ⊕ ⊕ ⊕ ⊖ moderate
Ziegler AG et al. [33]	FDR	0–17 y f/up: 5.2 y (median)	The IAb incidence (95% CI) was (per 1000 person‐y): 9 m: 18.5 [12.7, 27] 2 y: 21 [15.9, 29] 5 y: 9.1 [6.3, 12.5] 8 y: 9.2 [5.7, 11.7] 11 y: 5.1 [3.1, 8.5] Early peak seroconversion incidence was most evident in children with high‐risk HLA DR3/4‐DQ8 or DR4/4‐DQ8 genotypes	46% for seroconverters within 10 y 54% for seroconverters at age 2 y 31% for seroconverters after age 2 y	n.a.	Prevalence data and predictors ⊕ ⊕ ⊕ ⊕ high
Koskinen MK et al. [34]	High‐risk all IAb+ with at least 1 IVGTT	3 m‐15 y f/up: median 12.02 y for non‐progressors, 5.49 y for progressors	52.1% were IAb+ at the time of first IVGTT Multiple IAb inversely correlated with FPIR (*p* < 0.0001)	30.3% of children developed Stage 3 T1D Median age at diagnosis 6.58 y (range 1.63–16.11) Youths with multiple IAbs almost invarIAbly developed Stage 3 T1D over time	n.a.	Prevalence data and predictors ⊕ ⊕ ⊕ ⊖ high
Sosenko JM et al. [35]	High‐risk population	Progressor: 9.9 ± 6.4; Non‐progressor:12.7 ± 8.9 f/up: At least 3 y prior to diagnosis	n.a.	Progressors had lower baseline FPIR values (adjusted for age and BMI) than non‐progressors. They have a greater decline in the FPIR from 1.5 to 0.5 y before diagnosis than from 2.5 to 1.5 years before diagnosis of Stage 3 T1D	n.a.	Prevalence data and predictors ⊕ ⊕ ⊕ ⊖ moderate
Helminen O. et al. [36]	High‐risk population (HLA+ and IAb+)	Newborn‐15 y, f/up: until 15 y or T1D onset	n.a.	45% progressed to Stage 3 T1D OGTT 2 h values started to be consistently higher in the progressors 1.5 y before diagnosis (*p* < 0.001)	n.a.	Prevalence data ⊕ ⊕ ⊕ ⊖ moderate Predictors ⊕ ⊕ ⊕ ⊕ high
Ismail HM et al. [37]	High‐risk population	Progressors: 10.5 y (median) Non‐progressors: 12.7 y f/up: 3.8 ± 2.7 y	n.a.	35.9% progressed to Stage 3 T1D The risk of Stage 3 T1D is increased with lower early C‐peptide response and higher late C‐peptide responses	n.a.	Prevalence data and predictors ⊕ ⊕ ⊕ ⊖ moderate
Koskinen MK et al. [38]	High genetic risk	4.6 y F/up: 2.7 y	n.a.	FPIR was decreased as early as 4–6 y before the diagnosis of Stage 3 T1D	n.a.	Predictors ⊕ ⊕ ⊕ ⊖ moderate
O'Rourke C et al. [39]	FDR with ≥1 IAb+	17 ± 13 y f/up: 18 y	n.a.	14% (of whom 6% with single IAb+ vs. 27% with multiple IAb+) developed Stage 3 T1D	n.a.	Prevalence data ⊕ ⊕ ⊕ ⊕ high
So M et al. [40]	FDR with multiple IAb+ without clinical T1D	0–18 y f/up 2.1 y ± 2.3 (for Maintainer) 5.2 y ± 2.8 (for Reverter)	n.a. Reversion: 4.1%	Estimated cumulative 5‐y risk of Stage 3 T1D: R 11% M 42% No significant difference in Stage 3 T1D incidence between the R who regained their multiple IAb+ status and those who did not	n.a.	Prevalence data ⊕ ⊕ ⊕ ⊖ moderate
Truyen I et al. [41]	High‐risk population (FDR with IAb+)	IAb+: 14 y (8–23) IAb−: 13 y (6–24) f/up: median 5.1 y	n.a.	46 (8.2%) FDR IAb+ developed Stage 3 T1D Random proinsulin/C‐peptide ratio was an independent predictor of the risk of Stage 3 T1D (*p* ≤ 0.001)	n.a.	Prevalence data and predictors ⊕ ⊕ ⊕ ⊖ moderate
Vehik K et al. [42]	High‐risk	<10 y f/up: 15 y	7% IAb+ Reversion 19–29%, in single IAb+ 85% of the reversion of single IAb occurred within 2 y of seroconversion and was associated to HLA genotype, decreasing titre and age	28% of the IAb+ developed Stage 3 T1D	n.a.	Prevalence data and predictors ⊕ ⊕ ⊕ ⊖ moderate
Ziegler AG et al. [44]	High‐risk/ FDR	0–15 y f/up 20 y	n.a.	Risk of progression to Stage 3 T1D in 10 y: multiple IAbs: 69.7% single IAb: 14.5% no IAb: 0.4%	n.a.	Predictors ⊕ ⊕ ⊕ ⊕ high
Bingley PJ et al. [45]	FDR with ICA+	3–40 y f/up 4.95 y (median)	n.a.	29% developed Stage 3 T1D within 5 y of f/up 5 y Stage 3 T1D risk: ≤ 10 y age: 59%, ≥25 y age: 62% Independent determinants were age, FPIR; baseline impaired glucose tolerance, number of additional IAb markers, but not IAb type or genotype.	n.a.	Prevalence data and predictors ⊕ ⊕ ⊕ ⊖ moderate
Kwon BC et al. [46]	High‐risk/ FDR IabIAb+	< 16 y f/up: 15 y	n.a.	30% with 2 IAb+ developed Stage 3 T1D Undiagnosed participants more frequently had low IabIAb levels and later appearance of IabIAb than diagnosed participants	n.a.	Prevalence data and predictors ⊕ ⊕ ⊕ ⊖/⊕ ⊕ ⊕⊕ ⊕ ⊕ ⊕ ⊖ moderate
Winkler et al. [47]	FDR vs. general population	<15 y f/up: up to 15 y	n.a.	n.a.	3.6% in screened vs. 28.7% in no screened developed DKA	Prevalence data ⊕ ⊕ ⊕⊖/⊕ ⊕ ⊕ ⊕ moderate/high
Elding Larsson et al. [48]	High risk who developed T1D	From 3 m of age f/up: up to 15 y	n.a.	n.a.	8% developed DKA Multiple seroconversion (3 IAb) was associated with the most rapid development of T1D (HR = 4.52, *p* = 0.014)	Prevalence data and predictors ⊕ ⊕ ⊕ ⊖ moderate
Triolo et al. [49]	High risk who developed T1D	11.4 y (median) vs. 15.25 y f/up: not reported	n.a.	n.a.	3.67% developed DKA	Prevalence data ⊕ ⊕ ⊕ ⊖ moderate
Elding Larsson et al. [50]	High‐risk	3 m‐15 y f/up: 15 y	n.a.	Stage 3 T1D was developed in 1.4% of children <5 y	15% DKA rate at onset of T1D in <2 y; 11.3% in <5 y	Prevalence data ⊕ ⊕ ⊕ ⊖ moderate
Ghalwash M. et al. [57]	High‐risk	2–6 y f/up: up to 15 y	n.a.	10% developed Stage 3 T1D by the age 15 y Sensitivity of 82% (95% CI 79–86) and PPV of 79% (95% CI 75–80) for Stage 3 T1D by age 15 y	n.a.	Prevalence data and predictors ⊕ ⊕ ⊕ ⊖/⊕ ⊕ ⊕ ⊕ moderate/high
Siljander HT et al. [58]	High‐risk	Newborn f/up: 6 y	n.a.	69% with >1 IAb+ developed Stage 3 T1D Progressors were younger at seroconversion, had higher levels of IAb, lower FPIR and HOMA‐IR, higher HOMA‐IR/FPIR A low FPIR (>24 mU/L) identifies future cases of Stage 3 T1D with high accuracy (5‐y progression rate >80%).	n.a.	Prevalence data and predictors ⊕ ⊕ ⊕ ⊖ moderate
Vehik K et al. [59]	High‐risk	11.1 (IQR 9.0–12.8 y) f/up: until 11.1 y or T1D onset	n.a.	33% developed Stage 3 T1D A relative increase ≥10% in HbA1c from baseline best marked the increased risk of Stage 3 T1D (73.6% sensitive; 88.3% specific; PPV 76%) and was as informative as OGTT 2‐hPG	n.a.	Prevalence data and predictors ⊕ ⊕ ⊕ ⊖ moderate
Xu P et al. [60]	FDR IAb+	11 y (median), 13 y, 17 y f/up: median 2 y	n.a.	Stage 3 T1D developed in a total of 414 youths with dysglycaemia (28.7%) during further f/up F/up time between dysglycaemia and Stage 3 T1D was 1.8 y (IQR 0.8–3.6) The overall 5‐y risk was 42% Progression to dysglycaemia was associated with: IA‐2A titres, and 2‐h glucose and fasting C‐peptide Progression to Stage 3 T1D was associated with the number of IAb+, peak C‐peptide level, HbA1c level, and age	n.a.	Prevalence data ⊕ ⊕ ⊕ ⊖ moderate Predictors ⊕ ⊕ ⊕ ⊕ high
Redondo MJ et al. [61]	High‐risk IAb+	11.2 y (1.7–46.6) f/up: median 3.6 y	n.a.	68% developed Stage 3 T1D Participants with the Index60 ≥ 2.00 had more typical characteristics of Stage 3 T1D than participants with 120’ BG >11.1 mmol/L	n.a.	Prevalence data and predictors ⊕ ⊕ ⊕ ⊖ moderate
Ismail HM et al. [62]	High‐risk	13.8 ± 9.6 y f/up: 3.8 ± 1.7 y	n.a.	10.5% of IAb+ progressed to Stage 3 T1D At OGTT, Glucose Response Curves changed from biphasic (two peaks) to monophasic (one peak) (75.4% vs. 51% respectively, *p* < 0.001) to monotonic (continuous increase) during the progression to Stage 3 T1D	n.a.	Prevalence data and predictors ⊕ ⊕ ⊕ ⊖ moderate
Steck AK et al. [63]	IAb+ participants with CGM data	Progressors: 13.9 ± 3.8 y Non‐progressors: 16.6 ± 4.5 y F/up: 2.1 y (median)	n.a.	8/23 progressed to T1D at a median age of 13.8 y The cut‐off of 18% time spent at >140 mg/dL had 75% sensitivity, 100% specificity and a 100% PPV for Stage 3 T1D prediction after 25.5 m (median)	n.a.	Prevalence data ⊕ ⊕ ⊖ ⊖ low Predictors ⊕ ⊕ ⊕ ⊖ moderate
Steck AK et al. [64]	High‐risk IAb+	1–17 y (median 11.5) f/up: median 6 m	n.a.	18% progressed to Stage 3 T1D at 4.5 y The risk of progression to T1D in 1 y was 80% in those with time >140 md/dL of >10% vs. 5% in participants with time >140 md/dL of <10%	n.a.	Prevalence data and predictors ⊕ ⊕ ⊕ ⊖ moderate
Ylescupidez A et al. [65]	High‐risk	16.8 y (median) f/up: 12 m	n.a.	CGM measures in multiple IAb+ individuals are predictive of Stage 3 T1D but less than OGTT‐derived variables	n.a.	Predictors ⊕ ⊕ ⊕ ⊖ moderate
Desouter AK et al. [66]	FDR IAb+	IAb+: 16.6 y (13.4–23.4)	n.a.	13/34 FDR developed Stage 2 T1D 17/34 FDR developed Stage 3 T1D after a median of 40 m Longitudinally: repeated CGM associated with HbA_1c_ were nearly as effective as OGTT in predicting Stage 3 type 1 diabetes Cross‐sectionally, in predictions of Stage 3 T1D, CGM metrics equalled OGTT measures. OGTT‐based multivariable models remained superior	n.a.	Predictors ⊕ ⊕ ⊕ ⊖ moderate
Wilson DM et al. [67]	FDR IAb+ vs. IAb−	16.8 y (median) F/up: 1 y	n.a.	30.5% of IAb+ progressed to Stage 3T1D Spending ≥5–8% of the time with glucose >140–160 mg/dL (*p* = 0.02) was a good predictor of progression to Stage 3 T1D	n.a.	Prevalence data and predictors ⊕ ⊕ ⊕ ⊖ moderate

*Note*: Quality of evidence: high ⊕ ⊕ ⊕⊕, moderate ⊕ ⊕ ⊕⊖, low ⊕ ⊕ ⊖⊖, very low ⊕ ⊖ ⊖ ⊖.

Abbreviations: ABIS, All Babies in Southeast Sweden; ICA512A, antibodies to ICA‐512; AUC, area under the curve; ASK, Autoimmunitiy Screening for Kids program; BG, blood glucose; CGM, continuous glucose monitoring; DIPP, Diabetes Prediction and Prevention Study; DiPiS, Diabetes Prediction in SKane Study; DPTRS, Diabetes Prevention Trial Risk Score; DPT‐1, Diabetes Prevention Trial–Type 1; DKA, diabetic ketoacidosis; DKA, diabetic ketoacidosis; DBS, dried blood spot; EPDS, Edinburgh Postnatal Depression Scale; FG, fasting glucose; FDR, first‐degree relative; FPIR, first‐phase insulin response; f/up, follow‐up; GAD65, GADA antibody; GADA, glutamic acid decarboxylase antibodies; GP, general population; HbA1c, glycated haemoglobin; HR, hazard ratio; HT, height; HOMA‐IR, homeostasis model assessment of insulin resistance; HLA, human leukocyte antigen; IFG, impaired fasting glucose; IGT, impaired glucose tolerance; IAA, insulin autoantibodies; IA‐2A, insulinoma antigen‐2 autoantibodies; IVGTT, intravenous glucose tolerance test; IAb, islet autoantibody; ICA, islet cell antibodies; M, maintainers; m, month; n.a., not assessed; OGTT, oral glucose tolerance test; PSI, Parenting Stress Index; PHQ‐9, Patient Health Questionnaire‐9; PPV, positive predictive value; QALY, quality‐adjusted life‐year; R, reverters; RBA, radiobinding assay; ROC, receiver operating characteristic; SAI, State Anxiety Inventory; TEDDY, The Environmental Determinants of Diabetes in the Young; PTP, TrialNet's Pathway to Prevention; T1D, type 1 diabetes; T1DI, Type 1 Diabetes Intelligence; WT, weight; y, year/s; ZNT8A, zinc transporter 8 antibody.

**TABLE 3:** Summary and grading of evidence for moderate‐to‐high‐quality studies for psychological outcomes.

References	Population	Age at the screening F/up	Anxiety/distress	Level of evidence
Ziegler AG et al. [15]	Healthy children screened and parents from DiMelli	1.75 to 5.99 f/up: 2.4 (1.0–3.2)	Stress ↑ at T0 in mothers of IAb+ vs. IAb− but declined after 12 m	Prevalence data ⊕ ⊕ ⊕ ⊖ moderate
Goldstein E et al. [50]	Parents of high‐risk children and ≥2 IAb+ vs. IAb− RCT intranasal insulin vs. placebo	Newborn f/up: 0.5 to 6.7 y (mean 2.9)	Stress indexes = in case and control With Stage 3 T1D diagnosis: higher stress than control subjects. Stress decreased with duration of the f/up Single parents, urban environment, unemployment and chronic illness in the family were associated with higher stress, as was maternal age (*r* = −0.115, *p* = 0.039) but not paternal age	Prevalence data and Predictors ⊕ ⊕ ⊕ ⊖ moderate
Johnson SB et al. [51]	High‐risk and IAb+ vs. IAb−	Before 4.5 m of age f/up: 1–6 y	SAI score >40 in 47% of mothers and 34% of fathers (> in FDR, basal anxiety, risk perception accuracy, two or more IA+ test, child being an ethnic minority) < score with longer f/up (*p* < 0.05)	Prevalence data and Predictors ⊕ ⊕ ⊕ ⊖ moderate
Melin J et al. [52]	Parents of children of the general population	At birth f/up: annually from 2 to 15 y	Anxiety in 7.1% (> mothers, risk perception accuracy, low education) Anxiety was higher in mothers of children positive for IAb (*p* < 0.001) and those perceiving their child had higher risk of Stage 3 T1D (*p* < 0.001) having an FDR with Stage 3 T1D (*p* < 0.001 in mothers and 0.004 in fathers) Anxiety was less in highly‐educated parents (*p* < 0.001)	Prevalence data and Predictors ⊕ ⊕ ⊕ ⊖ moderate
O'Donnell HK et al. [53]	High risk with IAb+ and their caregivers	5.6–14.7 y f/up: median 6.1 months	Anxiety in 74.4% (> in Hispanic, >in FDR, basal anxiety, multiple IAb+, low education) During f/up, after the first 2 monitoring visits, it decreased	Prevalence data and Predictors ⊕ ⊕ ⊕ ⊖ moderate
Roth R et al. [54]	Mothers of high‐risk subjects/FDR	Before 3 m of age f/up: 6 m	Maternal postnatal depression scores were in the normal range with an overall post‐depression prevalence rate of 9% (vs 10–15% worldwide). Anxiety (< German mothers, > in minority, higher maternal age, low education)	Prevalence data and Predictors ⊕ ⊕ ⊕ ⊖ moderate
Smith LB et al. [55]	High‐risk/FDR/general population	0–15 y f/up: up to 15 y	Increased monitoring behaviours after IAb+ notification Greater monitoring behaviours in mothers than in fathers, in older children, first‐born children, non‐ethnic minority children and FDR families compared to general population families	Prevalence data and Predictors ⊕ ⊕ ⊕ ⊖ moderate

*Note*: Quality of evidence: high ⊕ ⊕ ⊕⊕, moderate ⊕ ⊕ ⊕⊖, low ⊕ ⊕ ⊖⊖, very low ⊕ ⊖ ⊖ ⊖.

Abbreviations: DKA, diabetic ketoacidosis; EPDS, Edinburgh Postnatal Depression Scale; FDR, first‐degree relative; f/up, follow‐up; GP, general population; HLA, human leukocyte antigen; IAb, islet autoantibody; m, month; PSI, Parenting Stress Index; PHQ‐9, Patient Health Questionnaire‐9; QALY, quality‐adjusted life‐year; SAI, State Anxiety Inventory; T1D, type 1 diabetes; y, year/s.

**TABLE 4:** Summary and grading of evidence for moderate‐to‐high‐quality studies for costs. Conversion from euros to dollars on 14 October 2024 (1 euro = 1.09 dollars).

References	Population	Age at the screening f/up	Costs	Level of evidence
McQueen RB et al. [19]	General population	2‐17 y f/up: 3 y	Cost per child screened: $47 vs. 141 of routine screening Cost per case detected was $4700 vs. no screening and $14,000 for routine screening versus no screening	⊕ ⊕ ⊕ ⊖ moderate
Vehik K et al. [29]	FDR	1‐17 y f/up: 5.8 y	Cost per child screened: $134 Cost per child IAb+: $2436 Cost per participant positive after rescreening: $4931	⊕ ⊕ ⊕ ⊖ moderate
Vehik K et al. [30]	FDR	3‐18 y f/up: 5 y	Cost for a IAb screen (including all IAb) is $70	⊕ ⊕ ⊕ ⊖ moderate
Karl FM et al. [56]	General population	1.75–5.99 y f/up: n.a.	Cost per child screened: $30.8 Cost per child diagnosed with Stage 1 or Stage 2 T1D: $9974	⊕ ⊕ ⊕ ⊖ moderate

*Note*: Quality of evidence: high ⊕ ⊕ ⊕⊕, moderate ⊕ ⊕ ⊕⊖, low ⊕ ⊕ ⊖⊖, very low ⊕ ⊖ ⊖ ⊖.

Abbreviations: f/up, follow‐up; FDR, first‐degree relative; IAb; islet autoantibody; T1D, type 1 diabetes; y, year/s.

### 3.1 Primary outcome: Impact of screening on the frequency of IAb+, T1D, DKA at T1D onset and psychological outcomes

#### 3.1.1 IAb positivity

Positive islet autoantibody tests were confirmed in a second sample in the studies included for this review. IAb positivity was 0.7–7.2% in the general population^11^, ^12^, ^13^, ^18^, ^20^ and 0.9–23.4% in the high‐risk/FDR population.^21^, ^22^, ^23^, ^24^, ^25^, ^26^, ^27^, ^28^, ^29^, ^30^, ^31^, ^32^, ^33^ The highest prevalence of IAb+ (52.1%) was observed in a Finnish study of high genetic risk.^34^ Differences in positive screening rates between programmes were influenced by the number of IAb+ screened (more IAbs tested led to higher IAb positivity rates), the assay used (variable specificity and sensitivity), IAb+ persistency (maintainer vs. reverter), variations in HLA genetic risk (higher rates in genetically at‐risk individuals) and the age of youths (optimal screening ages were 2, 5–7 and 10 years). The frequency of reversion from IAb+ to IAb− status was reported in only three studies and was relatively frequent for GAD65 (19–33%), insulin IAb (29%) and IA‐2A (57%)^12^, ^42^ but much rarer (4.1%) in IAb+ individuals in another cohort followed‐up for 5.2 years.^40^ Reversion was primarily restricted to children with single IAb positivity (24%), occurred within 2 years of seroconversion, and was associated with the HLA genotype (DQ2 vs. DQ8), decreasing IAb titres, and older age.^42^ In contrast, it was rare in children who developed multiple IAb+ (<1%).^2^ Among children in whom a single IAb+ reverted, 19–34% seroconverted back to positive.^40^, ^42^

#### 3.1.2 Diagnosis of stage 3 T1D through screening and progression during follow‐up

GADA and IA‐2A positivity at birth were associated with an increased risk of developing Stage 3 T1D (median age at Stage 3 T1D diagnosis: 8.8 [range 1–22] years), regardless of HLA risk genotypes and maternal diabetes.10 At 10 years of follow‐up, the highest risk was detected for IA‐2+ vs. GADA+ children (*p* = 0.048).^18^

In the general population, the prevalence of a Stage 3 T1D diagnosis detected at the time of the screening was 0.02–0.03%.^16^, ^20^ Instead, during follow‐up of Stage 1 and Stage 2 individuals, the proportion of IAb+ individuals who progressed to Stage 3 T1D varied based on age, follow‐up duration and number of IAb+, ranging from 17% to 53% of IAb+ children‐adolescents.^12^, ^18^ The cumulative risk of T1D at 10 and 18 years of follow‐up in people testing positive for multiple IAbs was 59.7% and 75.1%, respectively (*p* < 0.001),^18^ while the risk in people testing positive for a single IAb was 1.2% and 22.6%, respectively (p < 0.001).^18^ Fifty‐three percent of IAb+ individuals developed stage 3 T1D after a median of 8.6 years.^12^

In the high‐risk/FDR population, the prevalence of stage 3 T1D diagnosed by screening was 1% in first‐, second‐ and third‐degree relatives in one study.^32^ In high‐risk individuals, stage 3 T1D was diagnosed during follow‐up in 3.7–62% of IAb+ individuals, depending on IAb number, persistence, titre, sample size, age at the screening and follow‐up duration.^19^, ^21^, ^22^, ^23^, ^24^, ^28^, ^29^, ^33^, ^34^, ^35^, ^36^, ^37^, ^38^, ^39^, ^40^, ^41^, ^42^, ^43^, ^44^, ^45^, ^46^ In high‐risk/FDR individuals, the 10‐year risk of progression to Stage 3 T1D was 27–69.7% in people testing positive for multiple IAbs, 14.5–26% in people testing positive for one IAb and 0.4% in IAb− individuals.^24^, ^39^, ^44^ The IAb+ number predicted progression to Stage 3 T1D: four positive IAbs vs. one IAb had a hazard ratio (HR) of 33.1,^45^ while the presence of two IAbs had an HR of 1.85 (*p* = 0.04).^17^ The positive predictive value (PPV) of multiple IAb positivity was higher than HLA‐DQB1+ (PPV 61.1% vs. 23.7%).^18^, ^45^ In another study, the 15‐year risk of progression to stage 3 T1D varied from 18% to 88%, based on the stringency of IAb+ definition.^22^

Seroconversion of multiple IAbs was associated with an increased risk of T1D,^29^ and the most rapid development of T1D,^34^ and was inversely correlated with first‐phase insulin response (FPIR).^34^, ^35^

#### 3.1.3 Frequency of DKA and meta‐analysis

DKA occurred in 3.2% of general population screening programme individuals^16^ compared with 29–31% of those not enrolled in such programmes.^32^, ^47^ In the high‐risk/FDR population, DKA occurred in 3.3–8% of participants,^14^, ^16^, ^32^, ^48^, ^49^ with differences between studies in part attributable to different definitions of DKA used and other population demographics, as DKA was more common in younger children (15% in <2 years old) than in older children (11.3% in <5 years).^50^

**FIGURE 2:**
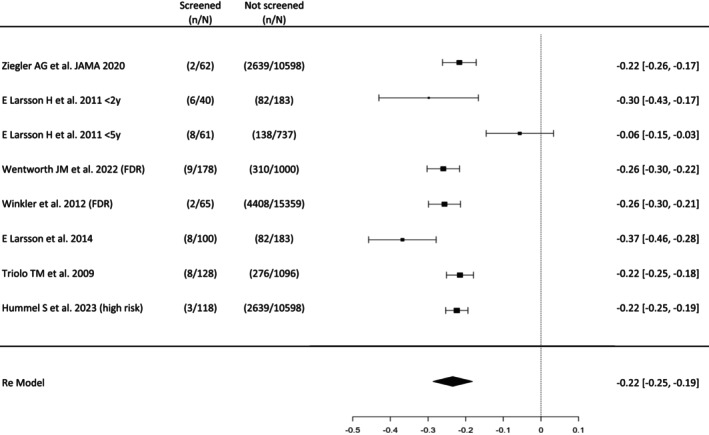
Forest plot showing the pooled difference in DKA risk in children‐adolescents screened for IAbs compared with those not screened. Data are reported as median and 95% CI. Measures of heterogeneity: Q(9) = 29.146, *p* < 0.001, tau^2^ = 0.0047, *I* ^2^ = 88.8%.

The impact of screening programmes on the frequency of DKA at Stage 3 T1D onset was assessed in seven studies, one divided into two sub‐studies based on the children's age (<2 years and <5 years).^50^ The observed risk differences ranged from −0.37 to −0.06, with 100% of estimates negative. Figure 2 shows the results of the meta‐analysis, and the mean risk difference estimated from the random‐effects model was −0.23 (95% CI: −0.29 to −0.18), with the average outcome differing significantly from zero (*z* = −8.73, *p* < 0.0001). The true outcomes were heterogeneous (Q(9) = 29.146, *p* < 0.001, tau^2^ = 0.0047, *I* ^2^ = 88.8%). A 95% prediction interval for the true outcomes was −0.3911 to −0.0530, with no evidence of outliers in the context of this model (studentized residuals ≤±2.8070), and no study was overly influential (according to Cook's distances). There was no funnel plot asymmetry (rank correlation *p* = 0.8618 and regression test *p* = 0.7152). In a subgroup meta‐analysis considering only studies in the general population, the findings were similar: according to the *Q*‐test, the true outcomes were heterogeneous (Q(5) = 24.9451, *p* = 0.0001, tau^2^ = 0.0078, *I* ^2^ = 92.0%).

No study reported data on morbidity or mortality related to DKA at Stage 3 T1D onset. Early diagnosis and reduced DKA rates led to a shorter hospitalization period at Stage 3 T1D onset in screened (11.4 days, 8.9–13.8) vs. non‐screened individuals (14.9 days, 14.4–15.3. *p* = 0.005).^47^ Screening programmes were associated with lower HbA1c^28^, ^32^ and BG levels at Stage 3 T1D onset^14^, ^32^ and with higher C‐peptide levels and reduced insulin requirements.^28^, ^32^ However, this significant benefit on HbA1c, BG, C‐peptide and insulin regimen was not sustained during the first 5 years following diagnosis.^47^

#### 3.1.4 Impact on psychological outcomes

Seven studies, reported in Table S3 described the psychological impact of screening.^16^, ^51^, ^52^, ^53^, ^54^, ^55^, ^56^ Two studies were conducted in the general population^16^, ^53^; one included the general population and FDR,^56^ whereas the other four included high‐risk youths or FDR.^51^, ^52^, ^54^, ^55^ Parental anxiety was assessed in all the studies using 2‐ to 12‐month questionnaires, and parents' risk perception with semi‐structured questions. The State–Trait Anxiety Inventory (STAI) measure was the most used, and a score of >40 was used to define probable clinical levels of anxiety.

Parental anxiety was detected in 7–74% of parents, and it was higher in parents of children with IAb+ at the time of metabolic staging.^51^, ^52^, ^53^, ^54^, ^55^, ^56^ Conversely, one study compared matched parents and detected no differences in stress indexes in parents of IAb+ children and controls.^51^

Anxiety appeared to wane over time, as the scores decreased after the second visit,^54^ after 12 months,^16^ and generally during follow‐up, persisting after 3 years in parents of children with multiple persistent IAb+.^52^, ^54^ Parental anxiety was higher in FDR with T1D, in mothers than in fathers, in parents of individuals with multiple IAb or persistent IAb, and was associated with higher maternal age, lower education status,^52^, ^53^, ^54^, ^55^ and accurate parent perceptions of the child's T1D.^52^, ^53^ Monitoring behaviours (such as BG checks) increased significantly in parents after IAb+ notification, continuing for up to 4 years, especially in mothers and FDR families compared with the general population.^56^

### 3.2 Secondary outcomes: Timings of screening and follow‐up, modality of follow‐up, metabolic monitoring and prediction of T1D onset

#### 3.2.1 Timing of screening

Only one study recommended annual screening starting in early childhood and continuing through early adolescence.^31^ By contrast, the other studies proposed IAb screening at specific landmark ages: at 2–3 years, shortly after the peak incidence of IAb; at 6 years, around the time of school entry; and in early adolescence, after which the risk of developing IAb significantly decreases.^21^, ^26^, ^33^

##### Risk for developing IAb+

The 5‐year risk of developing any or multiple IAb+ by screening at landmarked ages was reported only for the high‐risk/FDR population, and it decreased with age. It ranged between 2% and 8% for any IAb+ in individuals aged <2 years.^21^, ^26^, ^33^ At this age, one study reported a frequency of 6.3% for any IAb+ and of 4.3% for multiple IAb+ at 7.5 months of age.^21^ Around 6 years, the frequency of IAb+ was 3.2% for any IAb, 1.1% for multiple IAbs^21^ and 0.7–0.9% at 10–12.5 years.^26^, ^30^

##### Risk for developing Stage 3 T1D

Screening at 2 and 6 years provided a sensitivity of 82% and a PPV of 79% for Stage 3 T1D by the age of 15 years^57^; screening at 10 years provided a sensitivity of 90% and a PPV of 66% for Stage T1D by the age of 18 years; and double screening at 10 and 14 years a sensitivity of 93% and a PPV of 55%.^23^ No single time point captured most cases of Stage 3 T1D occurring by 12 years of age. The optimal age for a single screening was 4 years due to the highest sensitivity achieved by screening at this age (37.2% of cases identified by a single screening visit between 3 and 5 years of age; 95% CI 32.0–42.6).^21^, ^33^ Screening at five or at 8 years had a similar median incidence of IAb+ (9.1 and 9.2 per 1000 person‐years),^33^ whereas, at 11 years, the incidence dropped to 5.1 per 1000 person‐years.^33^ For each one‐year increase in age, the risk of any IAb seroconversion decreased by 5%, and most children (75%) seroconverted by 13 years of age.^14^, ^31^

#### 3.2.2 Timing of follow‐up

The timing and methods of follow‐up of the screening programmes varied. Some authors recommended monitoring stage 1 (IAb+) individuals with blood tests every 6 months and stage 2 (with abnormal BG) individuals every three months.^14^ Other protocols considered age at enrolment <5 years as a risk factor for developing T1D (HR 3.21 within 5 years of follow‐up).^45^

Age was significantly associated with time from seroconversion to onset of multiple and persistent IAbs and progression to stage 3 T1D.^22^ Consequently, metabolic monitoring in a few studies was conducted every 3 months until the age of four and subsequently every 3–12 months until age 15, depending on IAb positivity.^21^, ^24^, ^27^, ^42^, ^44^, ^48^, ^56^

#### 3.2.3 Modality of follow‐up, metabolic monitoring and prediction of T1D onset

HbA1c, random glucose levels, Oral Glucose Tolerance Test (OGTT), Intravenous Glucose Tolerance Test (IVGTT) and CGM were used to monitor IAb and dysglycaemia.

##### IAb positivity: type, level, single/multiple, persistence

IAA was the earliest IAb seen in children with a single IAb, with a sharp peak at age nine months^24^, ^33^ and a decline over the following five years.^27^, ^47^ High IAA levels were seen in predominantly multiple IAbs and at ages <3 years.^47^ GADA only increased until the second year and remained relatively constant.^27^, ^50^

Children‐adolescents with multiple persistent IAbs had higher progression to stage 3 T1D (*p* < 0.0001) compared with youths with non‐persistent positivity to >1 IAb occurring at different visits.^22^ Higher IAb titres also predicted progression to T1D.^58^

##### HbA1c, C‐peptide

In a few studies, a relative increase of HbA1c ≥10% from baseline predicted the risk of progression to T1D,^10^, ^49^, ^59^ while HbA1c remained normal in cases of recent‐onset hyperglycaemia.^49^ A decrease in C‐peptide within 6 months has been reported to be associated with progression to T1D, but it can be falsely low.^10^ The random proinsulin/C‐peptide ratio, a biomarker of beta‐cell function, was higher in IAb+ individuals, particularly those with multiple IAbs,^41^ and it was an independent predictor of the T1D risk and lower Homeostatic Model Assessment of Insulin Resistance (HOMA‐IR).^58^

##### OGTT

A few studies reported baseline data on OGTT in people with IAb+. Although some studies did not evaluate the relationship between OGTT and progression to Stage 3 T1D,^14^, ^39^, ^40^ another study suggested that OGTT results predict Stage 3 T1D onset.^61^ When OGTT was included in the follow‐up of IAb+, the test was performed every 6 to 12 months for T1D diagnosis and/or for T1D prediction (Table S4).^10^, ^28^, ^37^, ^62^ In one study, OGTT was performed every two years.^48^

OGTT diagnosed 30% of children who developed T1D aged <3 years, but only 5.7% after 3 years.^49^ Two‐hour glucose, peak C‐peptide and area under the curve (AUC) C‐peptide were of prognostic value for five‐year risk of progression to T1D,^16^ as well as impaired glucose tolerance (IGT), and it was an independent determinant of progression to T1D (120′‐blood glucose ≥7.8 mmol/L, HR 3.32).^45^ OGTT markers (2 h glucose, peak C‐peptide and AUC C‐peptide) significantly improved the prognostic accuracy compared with any index alone (*p* < 0.05).^60^, ^61^ Another metabolic marker, derived from OGTT, is Index 60, a composite measure derived from fasting C‐peptide, 60‐min C‐peptide and 60‐minute glucose levels during the OGTT. Youths with an Index 60 ≥2.00 had more typical characteristics of T1D than participants with blood glucose at 120′ >11.1 mmol/L and had a four‐year cumulative T1D risk of 95%.^61^

Lower early C‐peptide response (30–0′, C‐peptide difference) and higher late C‐peptide responses (120–60 min) were associated with an increased risk of T1D.^37^, ^62^ Glucose response curves changed from biphasic (due to the first and second phases of insulin secretion) to monophasic to monotonic (continuous increase) during progression to T1D.^37^ IVGTT data of IAb+ youths were reported in five studies^34^, ^35^, ^45^, ^58^ IVGTT improved the prognostic accuracy of 5‐year progression to T1D; in particular, lower first‐phase insulin response (FPIR) (<10th percentile) identified youths at T1D risk with high accuracy (five‐year progression rate >80%, HR 2.94),^38^, ^45^, ^58^ and a greater decline in FPIR was seen between 1.5 and 0.5 years before diagnosis.^35^ FPIR and higher HOMA‐IR/FPIR were of prognostic value for 5‐year risk T1D progression.^58^

##### CGM

CGM data in IAb+ were evaluated in seven studies,^17^, ^63^, ^64^, ^65^, ^66^, ^67^, ^68^ five of moderate‐to‐high quality (Table 2).^63^, ^64^, ^65^, ^66^, ^67^ Duration of blinded CGM was 5–10 days; data only at baseline were considered in one study^68^ and every 3–6 months in the others.^17^, ^63^, ^64^, ^65^, ^66^, ^67^ In three studies, the accuracy of CGM metrics to predict Stage 3 T1D was compared with OGTT results.^65^, ^66^, ^67^

Considering CGM metrics, a higher time spent with glucose values >140–160 mg/dL and increased mean glucose and glycaemic variability were found in progressors compared with non‐progressors.^63^, ^64^, ^65^ In a few studies, the cut‐off of 18% of time above (TA) 140 mg/dL had 75% sensitivity, 100% specificity and a 100% PPV for T1D prediction after 25.5 months (median).^64^ The cut‐off of 10% of time >140 mg/dL had 88% sensitivity, 91% specificity and a 67% PPV for T1D prediction after 1 year.^63^ A recent study detected TA 140 mg/dL >4.3% in rapid progressors compared with 1.1% in non‐progressors to Stage 3 T1D (*p* < 0.001).^66^

In one study, a comparison of CGM metrics with OGTT results revealed that CGM could help to identify individuals likely to rapidly progress to stage 3 T1D, including those with normal OGTT.^67^ In another study, multivariable analysis demonstrated that OGTT‐derived variables were more discriminative than CGM metrics for identifying progressors.^65^ In a recent study, longitudinally repeated CGM, only if associated with HbA_1c_, was nearly as effective as OGTT in predicting stage 3 T1D.^66^

Findings from the 13 studies classified as low‐ or very low‐quality evidence are reported in Table S2.^10^, ^11^, ^13^, ^43^, ^65^, ^68^, ^69^, ^70^, ^71^, ^72^, ^73^, ^74^, ^75^

## 4 DISCUSSION

This systematic review summarizes current evidence on the impact and value of IAb screening programmes in the general paediatric and high‐risk/FDR populations, along with evidence‐based follow‐up modalities (Table 5).

**TABLE 5:** Summary of the key findings.

	General population	High risk/FDR population
Impact of the screening		
Frequency of IAb+	0.7–7.2%	0.9–23.4%
Cumulative risk of progression to Stage 3 T1D		
In multiple IAbs+	At 10 y: 59.7%; 18 y: 75.1%	At 10 y: 27–69.7%
In single IAb+	At 10 y: 1.2%; 18 y: 22.6%	At 10 y: 14.5–26%
Prevalence of Stage 3 T1D in IAb+		
At the time of the screening	0.02–0.03%	1%
During f/up	17–53%	3.7–62%
Frequency of DKA at T1D onset	3.2%	3.3–8%
Psychological outcome (parental anxiety)	In 7.1% (one study) and declined after 12 m	In 34–74.4% and declined within 3 y
Modality of f/up		
Timings of screening	2, 6 and 10 y	
Timing of f/up	Every 3 m until the age of 4 y and subsequently Every 3–12 m until age 15, depending on IAb positivity	
Monitoring modalities	FBG, IAb, HbA1c and OGTT (every 6–12 m or for decision on insulin initiation, disease‐modifying therapy, or inclusion in trials) If possible 7–14 days CGM every 3–12 m Educate for symptoms and capillary glucose testing Psychological Support	

Abbreviations: CGM, continuous glucose monitoring; DKA, diabetic ketoacidosis; f/up, follow‐up; FBG, fasting blood glucose; HbA1c, glycated haemoglobin; IAb, islet autoantibody; m, month; OGTT, oral glucose tolerance test; T1D, Type 1 diabetes; y, year.

In response to the primary outcome, moderate‐to‐high‐quality evidence suggests that IAb positivity at the screening ranges between 0.7 and 23.4%,^11^, ^12^, ^13^, ^18^, ^19^, ^20^, ^21^ and in people testing positive for multiple IAb, the 10‐year risk of progression to Stage 3 T1D is between 27 and 69.7%.^24^, ^39^, ^44^ Early diagnosis of IAb+ provides time to educate families on symptoms of T1D onset and avoiding DKA. Meta‐analysis of these results confirmed a 23% average reduction in DKA in screened compared with unscreened individuals.^14^, ^16^, ^32^, ^48^, ^49^ Screening in the general population and follow‐up to age 18 was estimated to be cost‐saving in the USA if it reduced the DKA rate by 20% and lifetime HbA1c by 0.1%,^20^ whereas cost‐effectiveness studies in Europe are lacking. On the other side, screening appears to have an initial negative impact on psychological outcomes; however, this anxiety appeared to wane over time, persisting after 3 years in the parents of children with multiple persistent IAb+.^52^ Having a multidisciplinary team that includes a psychologist, taking the right time for communication, education and ensuring the adequate follow‐up, are fundamental organizational tools to control this outcome.

In response to the secondary outcomes, evidence suggests screening of IAb at landmark ages 2, 6 and 10 years, having a higher PPV for T1D.^23^, ^57^ Metabolic monitoring could be performed every 3 months for up to 4 years and then every 3–6 months until 15 years, depending on IAb positivity (titre, multiple, persistent).^21^, ^27^, ^33^, ^42^, ^48^, ^56^ The type of IAb, titre, single or multiple IAb+, persistence and individual age predicted progression to T1D.^29^ Knowing this data is important for estimating the organizational and economic impact when setting up a screening programme.

IAb and HbA1c monitoring should be integrated with OGTT, as in clinical practice, two‐hour glucose values contribute to the diagnosis of dysglycaemia (IGT), and the combination of markers derived from OGTT can improve the prognostic accuracy of progression to T1D and the timing of follow‐up according to a few studies.^16^, ^61^ Children with multiple IAb+ or progressing from one to multiple IAb+ should receive follow‐up every 3 months with HbA1c, fasting BG and annual OGTT and IAb, as previously reported in consensus reports.^5^, ^6^, ^7^, ^8^, ^9^, ^10^ CGM is now commonplace, and it may be useful for assessing risk of progression to T1D, including in those who are OGTT negative.^67^ The metric time above (TA) 140 mg/dL for more than 10–18% was associated in a few studies with a 1–5‐year T1D risk.^63^, ^64^ We expect to see further studies on the use of CGM in IAb+, as this technology can easily be repeated every 3–12 months, and it is cost‐effective when considering direct and indirect costs. Particularly in children under 6 years of age, CGM is certainly better tolerated by the child than OGTT and has less organizational burden on healthcare systems.

This review is strengthened by focusing on the impact of T1D screening in the paediatric age group, the application of GRADE to quality‐assess the evidence for selecting the best evidence, and our meta‐analysis of the impact of autoantibody screening on DKA risk.

However, it is limited by (i) lack of data from non‐White populations; (ii) the exclusion of screening programmes measuring only one IAb, as one IAb+ can commonly persist; the inconsistent testing for IAA, inclusion of the subjective test ICA in some studies; the lack of inclusion of ZnT8 antibodies in most studies, thereby underestimating the prevalence of screening‐detected IAb+; different islet autoantibody testing platforms perform differently; (iii) only a few current studies on CGM, which were only applied for 5–10 days. Differences in populations included, follow‐up modality and duration limited the ability to synthesize data from multiple studies. Thirteen studies also reported sources of bias, so they were classified as low‐ or very low‐quality evidence and, therefore, were not included.

In conclusion, this systematic review confirms that screening for IAbs and follow‐up in the general population and FDR/high‐risk individuals is valuable because it reduces the risk of DKA in mean by 23%. It brings evidence for the recommendations reported in Table 5, in terms of timings of screening, timing of follow‐up, and monitoring modalities. Moreover, screening programmes allow participation in trials with C‐peptide‐preserving drugs, a topic outside the aim of this review.

## AUTHOR CONTRIBUTIONS

Valentino Cherubini, Roberto Franceschi, Enza Mozzillo, Marco Marigliano and Giulio Maltoni conceptualized the systematic review/meta‐analysis. Valentino Cherubini, Roberto Franceschi, Enza Mozzillo, Marco Marigliano, Giulio Maltoni, Ernesto Maddaloni and Raffaella Buzzetti contributed to the design of the systematic review/meta‐analysis, contributed to the search strategy and drafted the manuscript. Luca Bernardini, Federica Ciambrelli, Alessandro Fierro, Evelina Maines, Francesca Di Candia, Ludovica Fedi, Andrea Scozzarella, Antonio Iannilli, Valentina Tiberi, Claudia Piona and Letizia Leonardi conducted data extraction, contributed to Figure 1, Tables and Appendices. Riccardo Pertile designed and performed the statistical analyses, contributed to Figure 2 and to the graphical abstract. Riccardo Bonfanti, Dario Iafusco, Claudio Maffeis, Ivana Rabbone and Carlo Ripoli interpreted the data. All authors carefully revised the manuscript and approved the final version of this paper. Roberto Franceschi, Enza Mozzillo and Marco Marigliano had full access to all the data in the study and took responsibility for the integrity of the data and the accuracy of the data analysis.

## FUNDING INFORMATION

We have no role as a funding source to disclose.

## CONFLICT OF INTEREST STATEMENT

RF has received travel grants from MOVI and Medtronic. EMo has received payment for the Advisory Board from Sanofi; support for attending meetings and/or travel grants from MOVI, Theras and Abbott; payment for educational events from Medtronic. Other financial interests as research grants from MOVI. MM has received consulting fees from Theras, Novo Nordisk and Ypsomed. Payment for educational events from Medtronic. Travel grants from MOVI. GM has received consulting fees from MOVI and travel grants from MOVI. EMad has received consulting fees from PikDare, Eli Lilly, MTD, Novo Nordisk, AstraZeneca, Abbott, Guidotti, and travel grants from Abbott, Theras and Guidotti. Payment for Advisory Board from Abbott, Novo Nordisk and MSD. RA has nothing to declare. LB has nothing to declare. RBO has nothing to declare. FC has nothing to declare. FDC has received payment for Advisory Board from Sanofi. LF has nothing to declare. AF has nothing to declare. DI has nothing to declare. AI has received a Travel Grant from Pfizer. LL has nothing to declare. CM has received grants for research projects from Medtronic, MOVI and Sanofi; has received consulting fees from Sanofi, Abbott, Eli Lilly; travel grants from Sanofi. Has received payment for Advisory Board from Sanofi and Abbott. EMai has nothing to declare. RP has nothing to declare. CP has received consulting fees from Sanofi and travel grants from MOVI. IR has nothing to declare. CR has received consulting fees from Theras and travel grants from Medtronic; has received payment for Advisory Board from Sanofi. AS has nothing to declare. VT has nothing to declare. RB has received consulting fees from Eli Lilly, Sanofi, Novo Nordisk, AstraZeneca, Abbott and Boehringer Ingelheim. Payment for presentation/honoraria from Eli Lilly, Sanofi, Novo Nordisk, AstraZeneca, Abbott and Boehringer Ingelheim. Travel grants from Novo Nordisk. Payment for Advisory Board from Eli Lilly, Sanofi, Abbott and Novo Nordisk. VC has received consulting fees from Eli Lilly, AstraZeneca, MOVI and Menarini. Payment for presentation/honoraria from Sanofi, Novo Nordisk, Theras and Erbozeta. Travel grants from Novo Nordisk, Sanofi and Theras. Payment for Advisory Board from Novo Nordisk, Sanofi and Theras.

## CLINICAL TRIAL REGISTRATION

The protocol of this systematic review and meta‐analysis was registered in PROSPERO on 23 March 2024 with accession CRD42024523781.

## Supporting information


**Table S1:** List of studies excluded at full‐text screening stage, with brief reasons (No. 56).

**Table S2:** Literature analysis after PICOS selection: summary of the studies and evidence grading for each study that reported prevalence data and predictors of stage 2–3 T1D and follow‐up modality.

**Table S3:** Literature analysis after PICOS selection: summary of the studies and evidence grading for each study that reported prevalence data and predictors of psychological outcomes.

**Appendix S1:** Supporting Information.

**Appendix S2:** Supporting Information.

## Data Availability

RF, EM and MM have directly accessed and verified the data reported in the manuscript. All authors should confirm that they had full access to all the data in the study and accept responsibility for submitting it for publication.
