# Supplementary material for: Impact of screening programmes for type 1 diabetes in youth: A systematic review and meta‐analysis
Source: Diabet Med. 2026 Jan 31;43(5):e70236. doi: 10.1111/dme.70236 (PMC13074144; doi:10.1111/dme.70236)
Supplement: Supplementary file 1 — Table S1: List of studies excluded at full‐text screening stage, with brief reasons (No. 56). [file DME-43-e70236-s003.docx]

**Supplemental Table S1: List of studies excluded at full-text screening stage, with brief reasons (No. 56)**

We followed this priority in the analysis and for reporting the reason for the exclusion: population (studies on newly diagnosed T1D, LADA, individuals with celiac or other autoimmune diseases, APECED, etc.), age, outcome, review, letter-commentary-editorial, assay, number.

| **No.** | **Title** | **Authors** | **Citation** | **DOI** | **Reason of exclusion** |
| --- | --- | --- | --- | --- | --- |
| 1 | A combined risk score enhances prediction of type 1 diabetes among susceptible children | Ferrat LA et al. | Nat Med. 2020 Aug;26(8):1247-1255. | 10.1038/s41591-020-0930-4 | Outcome |
| 2 | Diagnostic Capabilities of Islet Autoantibodies in Children with New-Onset Type 1 Diabetes Mellitus and Healthy Siblings | Korneva KG et al. | Sovrem Tekhnologii Med. 2021;12(6):29-34. | 10.17691/stm2020.12.6.04 | Population |
| 3 | Autoantibodies against zinc transporter 8 further stratify the autoantibody-defined risk for type 1 diabetes in a general population of schoolchildren and have distinctive isoform binding patterns in different forms of autoimmune diabetes: results from the Karlsburg Type 1 Diabetes Risk Study | Baumann K et al. | Diabet Med. 2021 Feb;38(2):e14389. | 10.1111/dme.14389 | Outcome |
| 4 | The risk of progression to type 1 diabetes is highly variable in individuals with multiple autoantibodies following screening | Jacobsen LM et al. | Diabetologia. 2020 Mar;63(3):588-596. | 10.1007/s00125-019-05047-w | Population |
| 5 | Progression likelihood score identifies substages of presymptomatic type 1 diabetes in childhood public health screening | Weiss A et al. | Diabetologia. 2022 Dec;65(12):2121-2131. | 10.1007/s00125-022-05780-9 | Population |
| 6 | A Type 1 Diabetes Genetic Risk Score Predicts Progression of Islet Autoimmunity and Development of Type 1 Diabetes in Individuals at Risk | Redondo MJ et al. | Diabetes Care. 2018 Sep;41(9):1887-1894.. | 10.2337/dc18-0087 | Age |
| 7 | Ethnic differences in progression of islet autoimmunity and type 1 diabetes in relatives at risk | Tosur M et al. | Diabetologia. 2018 Sep;61(9):2043-2053. | 10.1007/s00125-018-4660-9 | Age |
| 8 | Prediction of type 1 diabetes using a genetic risk model in the Diabetes Autoimmunity Study in the Young | Frohnert BI et al. | Pediatr Diabetes. 2018 Mar;19(2):277-283. | 10.1111/pedi.12543 | Outcome |
| 9 | Who Is Enrolling? The Path to Monitoring in Type 1 Diabetes TrialNet's Pathway to Prevention | Sims EK et al. | Diabetes Care. 2019 Dec;42(12):2228-2236. | 10.2337/dc19-0593 | Age |
| 10 | Predictive power of screening for antibodies against insulinoma-associated protein 2 beta (IA-2beta) and zinc transporter-8 to select first-degree relatives of type 1 diabetic patients with risk of rapid progression to clinical onset of the disease: implications for prevention trials | De Grijse J et al. | Diabetologia. 2010 Mar;53(3):517-24. | 10.1007/s00125-009-1618-y | Age |
| 11 | Simplifying prediction of disease progression in pre-symptomatic type 1 diabetes using a single blood sample | Bediaga NG et al. | Diabetologia. 2021 Nov;64(11):2432-2444 | 10.1007/s00125-021-05523-2 | Outcome |
| 12 | The development and utility of a novel scale that quantifies the glycemic progression toward type 1 diabetes over 6 months | Sosenko JM et al. | Diabetes Care. 2015 May;38(5):940-2. | 10.2337/dc14-2787 | Outcome |
| 13 | Clinical care advice for monitoring of islet autoantibody positive individuals with presymptomatic type 1 diabetes | Hendriks AEJ et al. | Diabetes Metab Res Rev. 2024 Feb;40(2):e3777. | 10.1002/dmrr.3777 | Review |
| 14 | Progression from single to multiple islet autoantibodies often occurs soon after seroconversion: implications for early screening | Chmiel R et al. | Diabetologia. 2015 Feb;58(2):411-3. | 10.1007/s00125-014-3443-1 | Letter |
| 15 | Exploring Minimally Invasive Approach to Define Stages of Type 1 Diabetes Remotely | Kontola H et al. | Diabetes Technol Ther. 2022 Sep;24(9):655-665. | 10.1089/dia.2021.0554 | Outcome |
| 16 | Screening and Prevention of Type 1 Diabetes: Where Are We? | Simmons KM et al. | Clin Endocrinol Metab. 2023 Nov 17;108(12):3067-3079. | 10.1210/clinem/dgad328. | Review |
| 17 | Screening for Type 1 Diabetes in the General Population: A Status Report and Perspective. | Sims EK et al. | Diabetes. 2022 Apr 1;71(4):610-623. | 10.2337/dbi20-0054. | Review |
| 18 | . Feasibility and Validity of In-Home Self-Collected Capillary Blood Spot Screening for Type 1 Diabetes Risk | Sing ABE et al. | Diabetes Technol Ther. 2024 Feb;26(2):87-94. | 10.1089/dia.2023.0345 | Outcome |
| 19 | Understanding Providersâ€™ Readiness and Attitudes Toward Autoantibody Screening: A Mixed-Methods Study,,"Ospelt E., Hardison H., Rioles N., Noor N., Weinstock R.S., Cossen K., Mathias P., Smego A., Mathioudakis N., Ebekozien O.","(Ospelt E., eospelt@t1dexchange.org | Ospelt E et al. | Clin Diabetes. 2024 Winter;42(1):17-26. | 10.2337/CD23-0057 | Outcome |
| 20 | Historical Insights and Current Perspectives on the Diagnosis and Management of Presymptomatic Type 1 Diabetes | Simmons KMW et al. | Diabetes. Diabetes Technol Ther. 2023 Nov;25(11):790-799. | 10.1089/dia.2023.0276 | Commentary |
| 21 | Screening for Type 1 Diabetes Risk in Newborns: The Freder1k Pilot Study in Saxony | Hommel A et al. | Horm Metab Res. 2018 Jan;50(1):44-49 | 10.1055/s-0043-120921 | Outcome |
| 22 | Pancreatic islet autoantibodies as predictors of type 1 diabetes in the diabetes prevention trial-type 1 | Orban T et al. | Diabetes Care. 2009 Dec;32(12):2269-74. | 10.2337/dc09-0934 | Outcome |
| 23 | Risk of beta-cell autoimmunity presence for progression to type 1 diabetes: A systematic review and meta-analysis | Ling Q et al. | Autoimmun. 2018 Jan;86:9-18. | 10.1016/j.jaut.2017.09.012 | Review |
| 24 | Barriers to Screening: An Analysis of Factors Impacting Screening for Type 1 Diabetes Prevention Trials | Kinney M et al. | J Endocr Soc. 2023 Jan 11;7(3):bvad003. | 10.1210/jendso/bvad003 | Outcome |
| 25 | Lessons and gaps in the prediction and prevention of type 1 diabetes | Mameli C et al. | Pharmacol Res. 2023 Jul;193:106792. | 10.1016/j.phrs.2023.106792 | Review |
| 26 | Progression to diabetes in relatives of type 1 diabetic patients: mechanisms and mode of onset | Ferrannini E et al. | Diabetes. 2010 Mar;59(3):679-85. | 10.2337/db09-1378 | Outcome |
| 27 | Progression of type 1 diabetes from latency to symptomatic disease is predicted by distinct autoimmune trajectories | Kwon BC et al. | Nat Commun. 2022 Mar 21;13(1):1514. | 10.1038/s41467-022-28909-1 | Outcome |
| 28 | Heterogeneity of DKA Incidence and Age-Specific Clinical Characteristics in Children Diagnosed With Type 1 Diabetes in the TEDDY Study | Jacobsen LM et al. | Diabetes Care. 2022 Mar 1;45(3):624-633. | 10.1089/dia.2019.0444 | Outcome |
| 29 | Parent preferences for delaying insulin dependence in children at risk of stage III Type 1 diabetes | DiSantostefano RL et al. | Diabetes Technol Ther. 2020 Aug;22(8):584-593. | 10.1089/dia.2019.0444 | Outcome |
| 30 | Nicotinamide protected first-phase insulin response (FPIR) and prevented clinical disease in first-degree relatives of type-1 diabetics | Olmos PR et al. | Diabetes Res Clin Pract. 2006 Mar;71(3):320-33. | 10.1016/j.diabres.2005.07.009 | Outcome |
| 31 | INnoVative trial design for testing the Efficacy, Safety and Tolerability of 6-month treatment with incretin-based therapy to prevent type 1 DIAbetes in autoantibody | Kero J et al. | Diabet Med. 2022 Oct;39(10):e14913. | 10.1111/dme.14913 | Outcome |
| 32 | Low-Dose Antithymocyte Globulin: A Pragmatic Approach to Treating Stage 2 Type 1 Diabetes | Foster TP et al. | Diabetes Care. 2024 Feb 1;47(2):285-289. | 10.2337/dc23-1750 | Outcome |
| 33 | The Deterrence of Rapid Metabolic Decline Within 3 Months After Teplizumab Treatment in Individuals at High Risk for Type 1 Diabetes. | Sims EK et al. | Diabetes. 2021 Dec;70(12):2922-2931. | 10.2337/db21-0519 | Outcome |
| 34 | What does the licensing of teplizumab mean for diabetes care? | Quinn LM et al. | Diabetes Obes Metab. 2023 Aug;25(8):2051-2057. | 10.1111/dom.15071. | Editorial |
| 35 | The importance of biomarker development for monitoring type 1 diabetes progression rate and therapeutic responsiveness | Fyvie MJ et al. | Front Immunol. 2023 May 15;14:1158278. | 10.3389/fimmu.2023.1158278 | Review |
| 36 | Unmethylated Insulin as an Adjunctive Marker of Beta Cell Death and Progression to Type 1 Diabetes in Participants at Risk for Diabetes | Simmons KM et al. | Int J Mol Sci. 2019 Aug 8;20(16):3857. | 10.3390/ijms20163857 | Outcome |
| 37 | One-Hour Oral Glucose Tolerance Tests for the Prediction and Diagnostic Surveillance of Type 1 Diabetes | Simmons KM et al. | J Clin Endocrinol Metab. 2020 Nov 1;105(11):e4094-101. | 10.1210/clinem/dgaa592 | Outcome |
| 38 | Adherence to oral glucose tolerance testing in children in stage 1 of type 1 diabetes: The TEDDY study | Driscoll KA et al. | Pediatr Diabetes. 2021 Mar;22(2):360-368. | 10.1111/pedi.13149 | Outcome |
| 39 | Early hyperglycemia detected by continuous glucose monitoring in children at risk for type 1 diabetes | Steck AK et al. | Diabetes Care. 2014 Jul;37(7):2031-3. | 10.2337/dc13-2965 | Outcome |
| 40 | Continuous glucose monitoring has an increasing role in pre-symptomatic type 1 diabetes: advantages, limitations, and comparisons with laboratory-based testing | Joshi K et al. | Clin Chem Lab Med. 2023 Jun 23;62(1):41-49. | 10.1515/cclm-2023-0234 | Number |
| 41 | The association of physical activity to oral glucose tolerance test outcomes in multiple autoantibody positive children: The TEDDY Study | Johnson SB et al. | Pediatr Diabetes. 2022 Nov;23(7):1017-1026. | 10.1111/pedi.13382 | Outcome |
| 42 | Comparison of Metabolic Outcomes in Children Diagnosed with Type 1 Diabetes Through Research Screening (Diabetes Autoimmunity Study in the Young [DAISY]) Versus in the Community | Chan CL et al. | Diabetes Technol Ther. 2015 Sep;17(9):649-56. | 10.1089/dia.2015.0029 | Number |
| 43 | Screening for asymptomatic β-cell autoimmunity in young children | Ziegler AG et al. | Lancet Child Adolesc Health. 2019 May;3(5):288-290. | 10.1016/S2352-4642(19)30028-8 | Review |
| 44 | Efforts to screen kids for type 1 diabetes multiply | Couzin-Frankel J | Science. 2024 Mar; 15;383(6688):1164-1165. | 10.1126/science.adp1963 | Editorial |
| 45 | IA-2A positivity increases risk of progression within and across established stages of type 1 diabetes | Sims EK et al. | Diabetologia. 2025 May;68(5):993-1004. | 10.1007/s00125-025-06382-x | Age |
| 46 | Feasibility and Validity of In-Home Self-Collected Capillary Blood Spot Screening for Type 1 Diabetes Risk | Sing ABE et al. | Diabetes Technol Ther. 2024 Feb;26(2):87-94. | 10.1089/dia.2023.0345 | Age |
| 47 | Enhancing Type 1 Diabetes Immunological Risk Prediction with Continuous Glucose Monitoring and Genetic Profiling | Montaser E et al. | Diabetes Technol Ther. 2025 Apr;27(4):292-300. | 10.1089/dia.2024.0496 | Age |
| 48 | Characteristics of autoantibody-positive individuals without high-risk HLA-DR4-DQ8 or HLA-DR3-DQ2 haplotypes. | Redondo MJ et al. | Diabetologia. 2025 Mar;68(3):588-601. | 10.1007/s00125-024-06338-7 | Age |
| 49 | Risk for progression to type 1 diabetes in first-degree relatives under 50 years of age. | Urrutia I et al. | Front Endocrinol (Lausanne). 2024 Aug 12;15:1411686 | 10.3389/fendo.2024.1411686 | Outcome |
| 50 | Identification of type 1 diabetes risk phenotypes using an outcome-guided clustering analysis. | You L et al. | Diabetologia. 2024 Nov;67(11):2507-2517. | 10.1007/s00125-024-06246-w | Outcome |
| 51 | Data-Driven Phenotyping of Presymptomatic Type 1 Diabetes Using Longitudinal Autoantibody Profiles | Ghalwash M et al. | Diabetes Care. 2024 Aug 1;47(8):1424-1431. | 10.2337/dc24-0198 | Outcome |
| 52 | Beyond Stages: Predicting Individual Time Dependent Risk for Type 1 Diabetes | Pribitzer S et al. | J Clin Endocrinol Metab. 2024 Nov 18;109(12):3211-3219. | 10.1210/clinem/dgae292 | Outcome |
| 53 | Novel Detection and Progression Markers for Diabetes Based on Continuous Glucose Monitoring Data Dynamics. | Montaser E et al. | J Clin Endocrinol Metab. 2024 Dec 18;110(1):254-262. | 10.1210/clinem/dgae379 | Outcome |
| 54 | Continuous glucose monitor metrics from five studies identify participants at risk for type 1 diabetes development. | Calhoun P et al. | Diabetologia. 2025 May;68(5):930-939. | 10.1007/s00125-025-06362-1 | Outcome |
| 55 | Early Dysglycemia Is Detectable Using Continuous Glucose Monitoring in Very Young Children at Risk of Type 1 Diabetes | Haynes A et al. | Diabetes Care. 2024 Oct 1;47(10):1750-1756. | 10.2337/dc24-0540 | Outcome |
| 56 | Costs of Public Health Screening of Children for Presymptomatic Type 1 Diabetes in Bavaria, Germany. | Karl FM et al. | Diabetes Care. 2022;45(4):837-844 | [10.2337/dc21-1648](https://doi.org/10.2337/dc21-1648) | Outcome |
