# Supplementary material for: Impact of screening programmes for type 1 diabetes in youth: A systematic review and meta‐analysis
Source: Diabet Med. 2026 Jan 31;43(5):e70236. doi: 10.1111/dme.70236 (PMC13074144; doi:10.1111/dme.70236)
Supplement: Supplementary file 2 — Table S2: Literature analysis after PICOS selection: summary of the studies and evidence grading for each study that reported prevalence data and predictors of stage 2–3 T1D and follow‐up modality. [file DME-43-e70236-s005.docx]

**Supplemental Table S2:** Literature analysis after PICOS selection: summary of the studies and evidence grading for each study that reported **prevalence data and predictors of stage 2-3 T1D and follow-up modality**.
Data are expressed as mean±SD, unless otherwise stated. The study of Raab et al. [15] is not included, as complete data are summarized in two subsequent studies [12,13].
High quality of evidence: ⊕⊕⊕⊕, Moderate: ⊕⊕⊕⊖, Low: ⊕⊕⊖⊖, Very Low: ⊕⊖⊖⊖.

| **Reference** | **Study design** | **Sample, age and comparator** | **Screening (Abs, age) and timing of follow up** | **Modality of follow-up Participation in trials** | **Prevalence data (IAb+, T1D, DKA)** | **Predictors of  stage 2-3 T1D** | **Study limitations, risk of bias,  level of evidence for prevalence data and for predictors** |
| --- | --- | --- | --- | --- | --- | --- | --- |
| **General Population** | | | | | | | |
| Knip M et al. [12] | Prospective | 3.475 youths from the general population  Age: 3-18 y Follow-up: 27 y Period: 1980-2007 Region: Finland | GADAs, IA-2A, ICA (all samples obtained in 1980 and 1986)  Cut-off: 5.36 RU (GADAs), 0.43 RU for (IA-2As)  GADA assay: disease sensitivity 82%, specificity  96%  IA-2A assay: disease sensitivity  72, specificity 100%  First sample in 1980, second sample in 1986 | IAb, visits | 34 individuals initially had GADAs (1.0%; 9 developed Stage 3 T1D) and 22 IA-2As (0.6%; 9 developed Stage 3 T1D). 7 individuals (0.2%) tested positive for both IAb.  Positive seroconversion rate over 6 years was 0.4% for GADAs and 0.2% for IA-2As, while the inverse seroconversion rates were 33% and 57%, respectively.  18 youths (53%) developed Stage 3 T1D after a median pre-diabetic period of 8.6 years (range 0.9 –20.3).  Among 6 participants who experienced an inverse seroconversion for GADAs by 1986, 2 progressed subsequently to Stage 3 T1D.  Over the observation period of 27 years, 12 male and 6 female individuals developed Stage 3 T1D (0.7 vs. 0.3%). The progressors were initially 1.7 years younger than the non-progressors (aged 9.1) vs. aged 10.8 years. | Initial positivity for GADAs and/or IA-2As had a sensitivity of 61% for T1D. Combined positivity for GADAs and IA-2As had both a specificity and a PPV of 100%  Both GADA and IA-2A positivity were associated with relatively high probability (26% and 41%, respectively) of T1D. Similarly, positivity for GADAs and/or IA-2As was related to an increased likelihood of progression to overt T1D.  All seven double-positive individuals developed clinical diabetes during the f-up. | IAA not available  Prevalence data  ⊕⊕⊕⊖ MODERATE  Predictors  ⊕⊕⊕⊖  MODERATE |
| Hummel S et al. [14] | Prospective | 169,446 children screened, and among them 128 children with Stage 3 T1D (Fr1da study) diagnosed with screening  vs 736 children with incident T1D (DiMelli cohort)  Name of the study: Fr1da  Age: 6.7 (5.0–9.1) vs 7.2 (4.5–9.2) yrs  Period: 2015-2022, vs 2009-2018  f/up: - (till developed stage 3)  Region: Bavaria | GADA, IA-2A and ZnT8A (3Screen ELISA  threshold of 25 U) | Educational training  Metabolic staging with HbA1c and OGTT (glucose, C-peptide)  Stage 2: every 3m  Stage 1: every 6m | 0.3% multiple IAb+ at the screening 27% of IAb+ developed Stage 3 T1D with a median of 2.3y (1.1.-4.3)  Youths followed-up in the screening, at the diagnosis of Stage 3 T1D had lower HbA1c (p<0.001), lower FG (p<0.05), and higher fasting C-peptide (p<0.001).  Fewer had ketonuria (p<0.001) or required insulin treatment (p<0.05) and only 2.5% presented with DKA. |  | F/up not clearly reported as IAb+ frequency  Prevalence data  ⊕⊕⊕⊖ MODERATE |
| Ziegler  AG et al. [16] | Prospective | 90.632 healthy children screened and 264 parents from DiMelli cohort for psychological outcomes    Age: 1.75 to 5.99  f/up: 2.4 (1.0-3.2) y  Period: 2015-2019  Region: Bavaria (Germany) | GADAs, IA-2A, ZnT8A (capillary) | Venous blood GADA, IA-2, ZnT8A, IAA (RBA)  OGTT for staging children with pre-symptomatic T1D  Blood gas analysis (pH) to detect stage 3 T1D | 280 (0.31%) children had Stage 1 or Stage 2 T1D, including 26 (0.03%) with Stage 3.  During f/up: other 36 children developed Stage 3 T1D.  The 3-y cumulative risk for Stage 3 T1D was 24.9% (54 cases; annualized rate, 9.0%).  DKA rate: 2/62 children (3.2% of Stage 3 T1D). | Factors associated with greater risk:  4 IAb+ vs 2 IAb+ (HR, 1.85; P = 0.04)  IA-2+ (HR, 3.4; P < 0.001), AUC and 60-minute OGTT results | Short f/up  Prevalence data  ⊕⊕⊕⊕ HIGH  Predictors ⊕⊕⊕⊕ HIGH |
| Till AM et al. [18] | Prospective | 11,986 school children screened from a general population  Age: 6–17 y  F/up: 18 y  Period 1995 and 1999  Region: Northeast Germany | GAD, IA-2A, IAA, ICA (RBA)  HLA-DQB1 | f/up of IAb+ for 18y:  examinations  HLA-DQB1 genotyping  For multiple or single high titer (>99^th^ percentile): annual f/up  All other children: bi-annual f/up | At the screening: 865 (7.2%) IAb+  At 1st f/up: 119 (77%) single IAb+ vs 36 (23%) multiple IAb+  Progression to Stage 3 T1D for 26 (17%) IAb+ children:  4 (15%) with a single IAb+, of whom 3 HLA-DQB1+; 22 (85%) with multiple IAb+, of whom 20 HLA-DQB1+ | The PPV of Stage 3 T1D was 61.1% for multiple IAb+ vs 23.7 % for HLA+  The cumulative risk for Stage 3 T1D at 10-and 18-y f/up: for multiple IAb+ was 59.7% and 75.1%, respectively (P<0.001)  For single IAb+ was 1.2% and 22.6%, respectively (P<0.001). Highest risk for IA-2+ at the 10-y f/up vs GADA + children (P=0.048) | Big sample size, long f/up  Prevalence data  ⊕⊕⊕⊕  MODERATE  Predictors ⊕⊕⊕⊕  HIGH |
| Gullstrand C et al. [19] | Prospective | 17,055 healthy children born between 1997 and 1999  Name of the study: ABIS Age: 0-5y F/up: 5 y  Period: 1998-2004  Region: Sweden | GADA, IA-2A HLA genotypes  f/up: 1, 2.5, 5 y of age | All the children IAb+ at any age and a subgroup of IAb-were HLA genotyped to study the association of HLA genotype with permanent and transient IAb | IAb+: 285/17.055 (1.7%) Stage 3 T1D: 32/17.055 (0.18%)  4/139 (2.9%) children GADA+  and 8/148 (5.4%) IA-2A+ at 2.5 y of age developed Stage 3 T1D before the age of 8 y    2/18 children (11.1%) positive for both GADA and IA-2A at 2.5 y of age developed Stage 3 T1D before the age of 8 y  GADA or IA-2A positivity at 5 y of age was associated with DR4-DQ8 haplotype and DR3-DQ2/DR4-DQ8 genotype | GADA+ or IA-2A+ give PPV of T1D: 3 and 5%, respectively  Both IAb+: give PPV of Stage 3 T1D of 11% | Short f/up, only 2 IAb.  Prevalence data  ⊕⊕⊕⊖ MODERATE  Predictors ⊕⊕⊕⊖ MODERATE |
| McQueen RB et al. [20] | Prospective | 10,029 children and adolescents, general population  Name of the study: ASK    Age: 2-17 y  f/up: January 2017 to 2020  Period: 2017-2020  Region: Denver, Colorado | IAA, GAD, IA2, ZnT8 measured with RBA and ECL (costs of TGA were excluded)  vs routine screening  vs usual care | F/up: if confirmed persistent IA | 0.48% of children developed multiple IAbs and 0.53 single IAb  86% of detected children did not have 1st degree relative with Stage 3 T1D |  | Partial and incomplete data, short f/up.  Prevalence data  ⊕⊕⊕⊖ MODERATE |
|  |  |  |  |  | **High risk/FDR** |  |  |
| Bonifacio E et al. [21] | Prospective | 8556 genetically at-risk children, of which 955 FDR  Name of the study: TEDDY  Age: before 4.5 months of age  Follow up: until 15 years of age  Period: 2004-2010  Region: USA and Europe | GADA, IA-2, IAA  Time of f-up:  every 3 m until 4 y of age every 3-6 months until 15 y of age | Landmark ages: 7.5m 2.125y 4.25y 6.25y 8.25y  f/up: 5 y from the landmark age | 809 (9.46%) developed IAb at median age of 3.2 y, of whom 471 developed multiple IAb  331 (3.7%) children developed Stage 3 T1D | The 5-year risk of developing any or multiple IAb was 6.3 and 4.3% respectively at landmark age 7.5m, 3.2 and 1.1% respectively at 6.25y (p < 0.0001)  Risk decay was similar between children FDR for Stage 3 T1D and children in the general population (p 0.44)  Risk decline was slight or absent in single IAA or GADA positivity  Influence of sex, HLA, and other susceptibility genes on risk subsided with increasing age and was abrogated by age 6 year (p<0.0001)  Highest sensitivity and PPV of multiple IAb phenotypes for Stage 3 T1D was achieved by IAb screening at 2 years and again at 5–7 years of age | Prevalence data  ⊕⊕⊕⊕  HIGH  Predictors ⊕⊕⊕⊕  HIGH |
| Frohnert BI et al. [22] | Prospective | 16,709 infant-toddlers with an increased risk of T1D  Name of the study: T1DI, from cohorts DIPP, BABYDIAB and BABYDIET, DiPiS, DaiSY and DEW-IT studies)  Age: ≤ 2.5 yrs  F/up: until 15 yrs of age or T1D onset (median 10.4 yrs of f/up)  Period: 1989-2012  Region: aggregated data from Finland, Germany, Sweden, and the U.S. | GADA, IAA, IA-2A  Timing of f/up intervals for assessing IAb varied among studies: range 3-36 m | GADA, IAA, IA-2A  Stage 3 T1D risk stratification based on a time closer to multiple IAb+ and on broader categories of multiple IAb | 865 (5%) developed >IAb during the whole follow-up  62% of multiple IAb+ progressed to Stage 3 T1D | The 15-y risk of progression to Stage 3 T1D varies markedly from 18 to 88% based on the stringency of IAb+ definition  Progression to Stage 3 T1D in multiple persistent IAb+ was significantly higher than other groups (p < 0.0001, not multiple or not persistent)  Age was significantly associated with time from seroconversion to multiple and persistent IAb and to Stage 3 T1D | Big sample size F/up intervals varied among individual studies.  Prevalence data  ⊕⊕⊕⊖/⊕⊕⊕⊕ MODERATE/HIGH  Predictors  ⊕⊕⊕⊕ HIGH |
| Ghalwash M et al. [23] | Prospective | 1890 patients with an increased T1D risk  Name of the studies:  DIPP, DiPiS, DAISY, DEW-IT, BABYDIAB  Age: 10-18 y  F/up: up to 18 y  or T1D onset (between 10-18y)  Region: Europe/USA | GAD, IA-2, IAA  Single screening at the age of 10 y or screening at two ages: 10 and 14 y | Anti-insulin, GAD, IA-2 at 10 or 14 y | 23.4% were positive for at least one Iab; 13.9% developed Stage 3 T1D | Screening at 10 y of age was highly effective to detect Stage 3 T1D by the age of 18y: sensitivity 90% (95% CI 86–95) with a PPV of 66%  Double screening at 10 and 14y was highly sensitive in detecting adolescents who will develop T1D (sensitivity of 93% with 95%, CI 89–97) but with lower PPV of 55% (49–60) | Big sample size  Age > 10 y and 14 y only  Prevalence data  ⊕⊕⊕⊖/⊕⊕⊕⊕ MODERATE/HIGH  Predictors ⊕⊕⊕⊖/⊕⊕⊕⊕ MODERATE/HIGH |
| Giannopoulou EZ et al. [24] | Prospective | 2441 pts with one parent with T1D or FDR  Name of the studies: BABYDIAB, BABYDIET  Age: 0-20 y  F-up: 20 yrs  Period: 1989-2000 (BABYDIAB); 2000-2006 (BABYDIET)  Region: Germany | IAA, GADA, IA2 and ZnT8A  At birth, 9 m and 2, 5, 8, 11, 14, 17 and 20 y  f/up: every 3 m until the age of 3 years and yearly until the age of 14 y | IAb  Visits | 218 (8.9%) children developed persistent Iab (150 had one Iab and 68 multiple Iab in the first positive sample).  Of the 150 children with one Iab at seroconversion, 56 (37%) developed multiple Iab during f/up  82 (41%) Iab+ children developed Stage 3 T1D during f/up  The earliest Iab development was seen in children with single IAA that progressed to multiple Iab or in those with persistent high-affinity single IAA, with a sharp peak in incidence observed at age 9 months  The peak incidence occurred at age 2 y for children who underwent seroconversion directly to multiple Iab and at 5 years for children who first seroconverted to GADA and subsequently developed other Iab | The 10-year risk of Stage 3 T1D was:  -56% (95% CI 38%, 74%) in children who had IAA and developed multiple IAb later  - 53% (95% CI 26%, 80%) in children who had GADA and developed multiple Iab later  -69% (95% CI 57%, 81%) in children who had multiple Iab in the first positive sample  In contrast, none of the 74 children with single low-affinity IAA or single GADA RBA-positive/ELISA-negative Iab developed Stage 3 T1D during f/up | Prevalence data  ⊕⊕⊕⊕  HIGH  Predictors ⊕⊕⊕⊕  HIGH |
| Helminen O et al. [25] | Prospective | 1162 youths IAb+  Name of the study: from the DIPP cohort (HLA positive at birth)  Age: since newborn  F/up: 4.5 – 6 y before diagnosis  Period: 1994-2014  Region: Oulu, Tampere, and Turku | ICA, IAA, GADA, IA2 | ICA, IAA, GADA, IA2  In IAb+: HbA1c, random BG (every 3 m)  OGTT (once a year)  every 3–12 m until the age of 15 y or T1D onset | 1162 (6.2% out of all included children) developed at least one IAb  A total of 335 children developed Stage 3 T1D  207 developed multiple IAb  229 a single IAb  370 ICA only  64 transient IAb | < HbA1c values in future progressors vs low-risk groups, at 6.0 to 4.5 y before their diagnosis or last visit. This finding, however, was not supported by BG or OGTT: no differences between the 2 groups   >incidence of dysglycemia in children progressing to Stage 3 T1D during f/up | Short f/up  Prevalence data  ⊕⊕⊕⊖ MODERATE  Predictors  ⊕⊕⊕⊖  MODERATE |
| Hoffman VS et al. [26] | Prospective | 2441 FDR from birth  Name of the study: BABYDIAB, BABYDIET  Age: at birth, 3.5, 6.5, 12.5 years  F/up: 6 y (short-term risk), 12y (mid-term risk) and 20y (long-term risk)  Period: 1989-2006  Region: Germany | GADA, IA-2, IAA, ZnT8  Visits and exams  at 9-12m 2 yrs  and every 3 years thereafter  Every 6 months if IAb + | GAD, IA-2, IAA, ZnT8 | The prevalence of IAb+ by the age of 20 years drops exponentially with age: was 8%, 4.6%, 2.6% and 0.9%, at the ages of birth, 3.5, 6.5 and 12.5 y, respectively | This risk decay model was consistent when the outcome was stratified for single or multiple IAb, or by HLA risk genotype  The short-term risks of Stage 3 T1D (within 6 years of f/up) at these landmark ages were 5.3%, 2.9%, 1.8% and 1%, respectively | Prevalence data  ⊕⊕⊕⊕  HIGH  Predictors ⊕⊕⊕⊕  HIGH |
| Krischer JP et al. [27] | Prospective | 8503/8676 genetically at-risk children enrolled  Name of the study: TEDDY study  F/up: up to 15 y of age  Period: 2004-2009  Region: USA | IAA, GADA and IA-2  Timing of f/up:  3 m up to 4 y of age 6 m thereafter | IAA, GADA and IA-2 | IAb were detected in 549 (6.5%). At 3 m 0.1% and at 6 m 0.2%  Of the 549, 43.7% had IAA only, 37.7% had GADA only, 13.8% had both GADA and IAA, 1.6% had IA-2 only and 3.1% had other combinations  The incidence of IAA only peaked within the first year of life and declined over the following 5 y, but GADA only increased until the second year and remained relatively constant | Participants who had GADA only were marginally less likely to develop Stage 3 T1D in the first year after seroconversion vs those who had IAA only (HR 0.35, 95% CI 0.12, 1.04, p = 0.06)  This protection was no longer seen after the second year (HR 0.92, 95% CI 0.51, 1.67, p = 0.78) | Prevalence data  ⊕⊕⊕⊕ HIGH  Predictors ⊕⊕⊕⊖ MODERATE |
| Mahon JL et al. [28] | Prospective | 12,636 FDR  Name of the study: TrialNet Natural History Story  Age: 1–45 y  F/up: 5 y  Period: 2004-2006  Region: USA, Canada, UK, Germany, Italy, Australia, and New Zealand | GADA, ICA512A, IAA (ICA tested if ≥1 another Ab+) | Youths with  ≥ 2 IAb+ in the 1st sample  or ≥1 Ab+ in the 2nd sample had a phase 2:  estimation of 5-y diabetes risk with OGTT every 6 m and IAb | 605 (4.8%) had ≥1 Ab+ in the 1st sample  Of 322 individuals enrolled in phase 2:  - 18 (5.6%) had Stage 3 T1D;  - 132 with estimated Stage 3 T1D risk/5 y <25% (normal OGTT and 1 IAb+ confirmed positive);  - 36 with estimated Stage 3 T1D risk/5 y ≥25% (normal OGTT and 2 Ab+ confirmed);  - 128 with estimated Stage 3 T1D risk/5 y ≥50% (IFG and/or IGT at OGTT or Normal OGTT+ ≥3 Ab+ confirmed) |  | Big sample size, Short f/up, frequency of OGTT every 6 m  Prevalence data  ⊕⊕⊕⊖/⊕⊕⊕⊕ MODERATE/HIGH |
| Ng K et al.  [29] | Prospective | 24,662 children at increased genetic or FDR  Name of the study: T1DI  Cohorts included: from DIPP (Finland), Germany (BABYDIAB), Sweden (DiPiS), and U.S. (DAISY and DEW-IT)  Age: 0-15 yrs  F/up: 15 years or until T1D diagnosis. Period: 1989-2012 Region: Finland, Germany, Sweden, and the U.S. | IAA, GADA, IA2 (RBA)  At age range:  1–2.0  2–3.0  3–4.0  4–5.0  5–10.0  and >10 years | IAb, visits | 1604 children with IAb+ (6,5%)  Among the 1604 IAb+ children, 600 (37.4%) developed Stage 3 T1D | 5-year Stage 3 T1D risks was different for IAb when stratified by quartiles of titer, ranging from 19% (GADA 1^st^ quartile) to 60% (IA-2A 4^th^ quartile)  Using the Iab type-specific titer thresholds in the 1481 children with all autoantibodies tested, the 5-year risk conferred by single (n = 954) and multiple (n = 527) Iabs could be stratified from 6 to 75% (P <0.0001)  Multivariable analysis confirmed the significance of associations between the 3 Iab titers and Stage 3 T1D risk | Big sample size, long f/up, solid study design.  IAb titers were measured using different assays across the study sites  Differences in the visit intervals of the study protocol  Prevalence data  ⊕⊕⊕⊖ MODERATE  Predictors  ⊕⊕⊕⊖ MODERATE |
| Vehik  K et al.   [30] | Prospective | 32,845 FDR  Name of the study: Trialnet Natural History Story  Age: 1–17 y  F/up: 5.8y  Period: 2004-2010  Region: USA, Canada, UK, Germany, Italy, Australia, and New Zeland | GAD65, ICA512, IAA | Annual rescreening was offered to IAb- | At the initial screen:  1,807 Ab + (5.5%) vs 31,038 (94.5%) IAb-  f/up of 31,038 Ab- children, seroconversion for: 205 to GAD65^+^, 155 to IAA, and 53 to ICA512^+^ | For each 1-y, the risk of seroconversion decreased by 11% for IAA [*P* < 0.0001] and 4% for GAD65 [*P* = 0.04]  The cumulative IAb seroconversion was 2% <10 y of age vs 0.7% ≥10 y of age  These data support annual screening for children <10 y of age and one additional screening in adolescence | Short f/up Bias of seroconversion rate because of no f/up on non-responders. D4  Prevalence data  ⊕⊕⊕⊖ MODERATE  Predictors ⊕⊕⊕⊖ MODERATE |
| Vehik K et al. [31] | Prospective | 42,447 participants FDR  (1.454 ICA+, 1,758 GAD65+, 899 ICA512+, 1094 IAA+)  n=11.813 IAb- from the population screened within the DPT-1 returned for rescreening  Name of the study: DPT-1  Age: 3-18 yrs F/up: 2.3 y (1.7-4.1)  Period: 1994-2002  Region: US | ICA, ICA512, GAD65, IAA  F/up:  individuals 3-10 y: re-screening annually;  individuals > 10 y: biennially | ICA, ICA512, GAD65  IAA was only measured on a small proportion of the youths at rescreening | At re-screening, seroconversion in 469 (4%) children  The median time to seroconversion was 2 y | The 2-year risk was highest in early childhood  For each 1-y increase in age in this cohort, the risk of any IAb seroconversion (HR 0.95, 95% CI 0.92–0.97) decreased by 5%, and for any two IAbs, the risk decreased by 13% (0.87, 0.82–0.93)  The majority of children (75%) seroconverted by 13 years of age  Annual screenings should be started  in early childhood and continued through early adolescence to identify the majority of individuals at risk for Stage 3 T1D and eligible for prevention trials | Big sample size/ Short f/up  Prevalence data  ⊕⊕⊕⊖/⊕⊕⊕⊕ MODERATE/HIGH  Predictors ⊕⊕⊕⊖ MODERATE |
| Wentworth JM et al. [32] | Prospective | 17,105 T1D relatives screened for IAb vs 7395 individuals with T1D in the general population   Name of the study: INIT  Age of the relatives: 15.7 (SD 10.8) Period: 2005-2019 Region: Australia and New Zealand  f/up: - | IAA, GADA, IA2  To Iab-: semi-annual HbA1c and OGTT  To Iab+: repeat Iab annually, while multiple Iab entered the study | Screening Iab, OGTT | Screened relatives:  652 (3.8%) had one Iab 306 (1.8%) had multiple Iabs 178 (1%) had Stage 3 T1D 9 (5%) had DKA, of whom 7 had not undertaken metabolic monitoring  General population: The frequency of DKA was 31% Age of diagnosis in the screened relatives was 13.4 (7.3) y vs 10.0 (5.8) y in the GP  HbA1c and insulin requirements following diagnosis were also lower in screened relatives, consistent with greater beta cell reserve  DKA occurred more frequently in who did not undertake OGTT monitoring |  | F/up duration not reported  Prevalence data  ⊕⊕⊕⊖ MODERATE |
| Ziegler AG et al. [33] | Prospective | First cohort: n=1650 children born to one FDR with T1D (BABYDIAB study)  Second cohort: n=150 children having both a FDR with T1D and T1D risk HLA genotypes (BABYDIET study)  F/up: BABYDIAB median 11.1 y (IQR 5.7–14.0 y); BABYDIET median 5.2 y (IQR, 3.2–6.6 years)  Period: 1989-2000  Region: Germany | IAA, GADA, IA-2, ZnT8  F/up of BABYDIAB cohort: visits scheduled at 9 m and at 2, 5, 8, 11, 14 and 17 y  Every 6 m in children IAb+  F/up of BABYDIET cohort: visits scheduled from the age of 3 m every 3 m until age 3 y and subsequently yearly | IAA, GADA, IA-2, ZnT8 | The islet autoantibody incidence (95% CI) was  9 m: 18.5 [12.7, 27] per 1000 person-y  2 y: 21 [15.9, 29] per 1000 person-years  5 y: 9.1 [6.3, 12.5] per 1000 person-y;  8 y: 9.2 [5.7, 11.7] per 1000 person-y  11y: 5.1 [3.1, 8.5] per 1000 person-y  The second cohort confirmed peak incidence around age 9 m and demonstrated an absence of seroconversion before this age  Seroconversion to IAA occurred earlier than  GADA, IA2 and ZnT8 (p<0.01) | Early peak seroconversion incidence was most evident in children with high-risk HLA DR3/4-DQ8 or DR4/4-DQ8 genotypes | Prevalence data  ⊕⊕⊕⊕  HIGH  Predictors  ⊕⊕⊕⊕ HIGH |
| Koskinen MK et al. [34] | Prospective | 574 children at high genetic risk with at least one IVGTT  Name of the study: DIPP  Age: median 4.6 y F/up: 2.7 y Period: from 1994 Region: Finland | IAA, GADA, IA-2A, ICA  IVGTT | IAb at 3 to 12 m intervals depending on age, IAb status, and study center  IVGTT after the detection of IAb | 268 were IAb+ (52.1%) at the time of first IVGTT (195 had multiple IAbs, 73 had single IAb)  133 children (30.3%) developed Stage 3 T1D during f/up at a median age of 8.6y (3.8-18.7) | Multiple IAbs inversely correlated with FPIR (p< 0.0001)  The HLA correlation with FPIR was secondary (p<0.0001) and was explained by association with IAb  Individuals with multiple IAbs almost invariably developed Stage 3 T1D over time. The progression rate to Stage 3 T1D is much lower in those with a single autoantibody  The effect of HLA DR-DQ risk genotypes may be related to the order in which the abs appear during the disease process | Short f/up  Prevalence data  ⊕⊕⊕⊖ MODERATE  Predictors  ⊕⊕⊕⊖ MODERATE |
| Sosenko JM et al. [35] | Prospective | 74 progressors to T1D with at least 1 FPIR measurement before the diagnosis  vs 270 non progressors  Name of the study: DPTT  Age  Progressor: 9.9±6.4  Non progressor:12.7±8.9  F/up: At least 3 years prior to diagnosis.  Period:- Region: USA, Canada | ICA, GAD65, IAA | IVGTT at baseline and at yearly intervals; performed after a minimum 10-h fast  The FPIR was defined as the sum of the insulin measurements at 1 and 3 min  OGTT was used for T1D diagnosis | 74 progressors had lower baseline FPIR values (adjusted for age and BMI) than non-progressors | Analysis of 26 progressors with annual FPIR for 3 y before diagnosis, revealed greater decline in the FPIR from 1.5 to 0.5 y before diagnosis than from 2.5 to 1.5 years before diagnosis | Prevalence data  ⊕⊕⊕⊖  MODERATE  Predictors ⊕⊕⊕⊖  MODERATE |
| Helminen O. et al. [36] | Prospective | 567 newborns with HLA susceptibility developed multiple IAb+  Name of the study: DIPP  Age: newborn-15 y F/up: until 15y or T1D onset Period: 1994-2014 Region: Turku, Oulu and Tampere | ICA, IAA, GADA, IA2 | OGTT and BG every 6 months | 255 (45%) progressed to Stage 3 T1D 312 (55%) remained non-diabetic Median diabetes-free survival time was 6.9 years Median time to Stage 3 T1D after observed  IFG was 5.2 y  after IGT, 0.7 y after random BG was 1y | OGTT 2 h values started to be consistently higher in the progressors 1.5 y before diagnosis (p < 0.001)  Dysglycemia detected with OGTT or random BG is useful marker in the prediction of time to onset of Stage 3 T1D in high-risk children | Sample size equal to cut-off  Prevalence data  ⊕⊕⊕⊖ MODERATE  Predictors  ⊕⊕⊕⊕ HIGH |
| Ismail HM et al. [37] | Prospective | 670 FDR IAb+  Name of the study: DPT-1  Age: 13.8 ± 9.6 y F/up: 3.8 ± 1.7 y Period: from 2000 Region: USA, Canada | GAD, IA-2A, IAA | 2-h OGTT (glucose-C-peptide) every 6 months for the diagnosis of Stage 3 T1D | 241 Youths progressed to Stage 3 T1D (35.9%) | Timing of C-peptide response during OGTT at baseline is predictive of Stage 3 T1D onset. ROC and regression analysis demonstrated that the risk of Stage 3 T1D is increased with lower early C-peptide response (30-0 min C-peptide difference) and higher late C-peptide responses (120–60 min C-peptide difference) (χ2 = 23.3, P < 0.001) | Short f/up  Prevalence data  ⊕⊕⊕⊖ MODERATE  Predictors ⊕⊕⊕⊖ MODERATE |
| Koskinen MK et al.  [38] | Retrospective | 685 children with at least one IVGTT performed after the initial appearance of IAb  n = 210 who had ≥2 IAb and progressed to T1D (progressors)  n = 192 who were ICA+ but non-progressors  Age: 3 m- 15 y  F/up: median 12.02 y for non-progressors, 5.49 y for progressors  Period: 1995-2013  Region: Finland | IAA, GADA, IA-2A  F/up: every 3 m | IVGTT  The recommended interval for  IVGTTs in IAb+ children was  every 6–12 months | n.a. (selection bias) | In the progressors, the first phase insulin response (FPIR) is decreased several years before the diagnosis of Stage 3 T1D; in particular it  Was decreased as early as 4–6 years before the diagnosis of Stage 3 T1D when compared to the non-progressors (p<0.001) | Retrospective  Predictors  ⊕⊕⊕⊖ MODERATE |
| O'Rourke C et al. [39] | Prospective | Screening of FDR 6193 individuals with ≥ 1 IAb+ (repeated)  Single IAb+ (N = 3790) vs Multiple IAb+ (N = 2403)  Name of the study: sub-cohort from the TrialNet's PTP study  Age: 17 ± 13 y  f/up: 18 y  Period: 2004 -2019  Region: USA, Canada, Germany, Italy, Sweden, Finland, Australia, New Zealand | IAA, GADA, IA-2A, ZnT8A | OGTT for Stage 3 T1D diagnosis, HbA1c  Models of progression to clinical diagnosis of Stage 3 T1D for pediatric (1-18y) and adult (18-35y) populations with single or multiple Ab+ | Stage 3 T1D rate: 868 (14%), of whom 229 (6%) with single IAb+ vs 639 (27%) with multiple IAb+  Over a 17-y monitoring period, a nominal 6 m of average undiagnosed time is achieved with:  17 visits for the pediatric single IAb+ population;  14 visits for the pediatric multiple IAb+ population; |  | Prevalence data  ⊕⊕⊕⊕ HIGH |
| So M et al. [40] | Prospective | 3284 multiple IAb+ without clinical T1D  Name of the study: subcohort from 201.617 FDR in TrialNet’s PTP Study  Age: 0- >18 y  F/up: 2.1 y ± 2.3 for Maintainer vs 5.2 y ± 2.8 for Reverter (loss of multiple- Ab+ to 1 or zero IAb+ on two occasions within 12 m)  Period: 2004-2019  Region: USA, Canada, Germany, Italy, Sweden, Finland, Australia, New Zealand | ICA, IAA, GADA, IA-2A, ZNT8  IAb+ confirmed on two occasions within 12 m | IAb, OGTT at baseline, HLA-DR3/DR4 | Reversion (R) occurred in 134 (4.1%)  The estimated cumulative 5-y risk of Stage 3 T1D:  for the R 11% (95% CI 6–18)  for the M 42% (95% CI 39–45) (P= 0.005).  During f/up, 41 (31%) of the R regained multiple- IAb+ status  No significant difference in T1D incidence (P = 0.99) between the R who regained their multiple IAb+ status (7/41, 17%) and those who did not (16/93, 17%) |  | Short f/up  Prevalence data  ⊕⊕⊕⊖ MODERATE |
| Truyen I  et al. [41] | Prospective | 561 FDR with IAb+ vs 561 age- and sex-matched persistently IAb- relatives  Age: IAb+: 14y (8-23) IAb-: 13y (6-24)  F/up: median 5.1 y  Period: 1989-2002  Region: Belgium | ICA, IA-2A, GADA, IAA | IAb+: were  genotyped for HLA  DQ  Random proinsulin, C-peptide and PI:C ratio were  determined | 46 (8.2%) FDR IAb+ developed Stage 3 T1D | At baseline IAb+ relatives had higher proinsulin/c-peptide ratio than IAb- or without later seroconversion (p<0.001)  In the presence of multiple or IA-2 antibodies, a proinsulin/C-peptide ratio exceeding percentile 66 of all antibody - relatives at entry (n = 784) conferred a 5-year diabetes risk of 50% and 68%, respectively (p<0.001 vs 13% for same antibody status with PI:C<percentile 66).  Random proinsulin/c-peptide ratio was an independent predictor of the risk of Stage 3 T1D (p≤0.001) | Short f/up  Prevalence data  ⊕⊕⊕⊖ MODERATE  Predictors ⊕⊕⊕⊖ MODERATE |
| Vehik  K et al.  [42] | Prospective | 8503 pts at genetic high-risk for T1D  Name of the study: TEDDY  Age: < 10 yrs  Follow-up: 15 yrs Period: 2004-2015 Region: USA, Finland, Germany, Sweden | IAA, GAD, IA2 (RBA)  F-up from 3 m to 15 y of age: WT, HT, IAb every 3 m until 4 years and every 3 or 6 months thereafter, depending on IAb positivity  If remission of all IAb occurs at any time during f-up for a period of 4 consecutive visits or 1 y, a f-up interval of 6 m becomes effective | Iab, visits | 596 pts (7%) developed ≥ 1 persistent IAb (37.8% remained single, 62,.2% developed multiple)  Median (IQR) age at initial seroconversion was 27.7 (15.2–48.3) m  28% of children (164 of 596) developed Stage 3 T1D, and by the time of diagnosis, 89% (146 of 164) were positive for ≥ 2 IAb at least once during f-up  Reversion was relatively frequent for GAD65 (19%) and insulin (29%) but was largely restricted to children who had a single IAb (24%) and rare in children who had developed multiple IAbs (<1%)  Of the children who reverted for their single IAb, 19% seroconverted back to positive | 85% of the reversion of single IAb occurred within 2 y of seroconversion and was associated to HLA genotype, decreasing titer and age | Big sample size, long f/up, frequency of f/up  Prevalence data  ⊕⊕⊕⊖ MODERATE  Predictors  ⊕⊕⊕⊖ MODERATE |
| Ziegler AG et al. [44] | Prospective | 13,377 children genetically at risk and FDR for T1D and parents  Names of the studies:  DAISY, DIPP, BABYDIAB9,  BABYDIET  Age: 0-15 y  F/up: 20 yrs  Period: 1989-2009  Region: USA, Finland, Germany | IAA, GAD65, IA2 and ZnT8 (in Colorado and Germany)  f/up every 3-6m and yearly after 24m | IAb, visits |  | Risk of progression to Stage 3 T1D in 10y  -with multiple IAbs (n = 585) was 69.7% (95% CI, 65.1%-74.3%) -with single IAb (n = 474) was 14.5% (95% CI, 10.3%-18.7%)  -with no IAb was 0.4% (95% CI, 0.2%-0.6%) by the age of 15 years  Progression to Stage 3 T1D in the children: FDR with multiple IAbs was faster for children with IAb seroconversion younger than age 3 years, HLA DR3/DR4-DQ8, and for girls | Predictors  ⊕⊕⊕⊕ HIGH |
| Bingley PJ et al. [45] | Prospective | 549 FDR of T1D patients with confirmed ICA+  Control population: 2860 schoolchildren  Name of the study: European Nicotinamide Diabetes Intervention Trial  Age: 3- 40 y (mean age 15.9 y)  F/up: 4.95 y (median)  Period: 1990- 1998  Region: 18 European countries, Canada, USA | Screening: ICA levels ≥20 JDF (Juvenile Diabetes Foundation Units) | At baseline: OGTT, IVGTT, GADA, IA–2A and IAA (RIA), and HLA genotype  OGTT at baseline and 6, 18, 30, 42, 54 and 60 m | 159 (29%) developed Stage 3 T1D within 5 y of f/up | Multivariate analysis: independent determinants were:  -Age (HR age at enrollment <5 y 3.21) -FPIR (HR FPIR <10th centile 2.94) - baseline impaired glucose tolerance (HR 120-min BG ≥7.8 mmol/l: 3.32)  -Number of additional IAb markers (HR ICA +3 additional Ab 33.10), but not IAb type or genotype.  5-y risk of Stage 3 T1D:  59% ≤10 y age, 11% ≥25 y age (*p*<0.0001), 62% FDR <25 y age with  ≥ 2 IAb+ at baseline  Screening and recruitment for future intervention trials should be limited to FDR <25 years, and should be based on IAb alone | Short f/up  Prevalence data ⊕⊕⊕⊖ MODERATE  Predictors  ⊕⊕⊕⊖ MODERATE |
| Kwon BC et al. [46] | Prospective | Among patients with HLA genotype+, or FDR with T1D, 2.145 participants who had 2 IAb+ at least once were included from early life  Name of the study: TIDI Study  Cohorts included: DAISY (U.S.), DEW-IT (U.S.), DiPiS (Sweden) and DIPP (Finland)  Age < 16 y F/up: 15 y Period: 1989-2012 Region: Finland, Sweden, Germany, U.S. | GADA, IAA, IA2 4 IAb-level groups by 5-year diabetes risk and 3 main autoimmune trajectories were identified:  predominantly multiple IAb (TR1), IAA (TR2), or GADA (TR3) as the first appearing autoantibodies  f/up: In IAb+: every 3 m, and after 24m yearly  Children with IAb- were followed less frequently | IAb, visits  In IAb+: Random BG, HbA1c, HT, WT | n = 643 (30%) progressed to Stage 3 T1D vs undiagnosed (n = 1502)  Median age 7.62 y in the diagnosed participants vs 12.87 in undiagnosed participants  In diagnosed participants, high IAA levels were seen in predominantly multiple IAb (traiectory TR1) and IAA (TR2) at ages <3 years, whereas IAA remained at lower levels in the undiagnosed  Different total duration of f/up at a given level differed between the 2 groups | Undiagnosed participants more frequently had low IAb levels and later appearance of IAb than diagnosed participants | Prevalence data  ⊕⊕⊕⊖ MODERATE  Predictors ⊕⊕⊕⊖ MODERATE |
| Winkler et al.  [47] | Prospective | N 101 IAb+ children with FDR vs 49,883 non screened children  Name of the study: BABYDIAB  Age <15 y  F/up: up to 15 y Period from 1995 Region Munich, Germany | GAD, IAA, IA-2, ZnT8 | DKA rate, HBA1c, length of hospitalization  5-y clinical course after Stage 3 T1D diagnosis (HbA1c, insulin dose) | At Stage 3 T1D onset, children who were followed after screening and were IAb+ had lower HbA1c (8.6 vs. 11%, p < 0.001), a lower prevalence of DKA (3.3 vs. 29.1%, p < 0.001), shorter hospitalization period at onset (11.4 vs. 14.9 d, p = 0.005  However, no differences between screened and non-screened children were observed with respect to HbA1c and insulin dose during the first 5 y after diagnosis |  | Small sample size  Prevalence data  ⊕⊕⊕⊖/⊕⊕⊕⊕ MODERATE/HIGH |
| Elding Larsson et al.  [48] | Prospective | Characteristics of 100 genetically at risk who developed T1D  Name of the study: TEDDY  Age: from 3 m  F/up: up to 15 y  Period: 2005-2011 Region: USA, Sweden, Finland, Germany | GADA, IA-2A, IAA | Visits every 3 m until 4 y  and then every 6 m until the age of 15 y  IAb+ continue to receive f/up every 3 months regardless of age  OGTT were performed every 6 m | Among the 100 individuals, 36% were asymptomatic at diagnosis  DKA was rare (8%)  An OGTT diagnosed 9/30 (30%) children above 3 y of age but only 4/70 (5.7%) below the age of 3 y  FDR had higher cumulative incidence than children from the general population (p < 0.0001) | Multiple seroconversion (3 IAb) was associated with the most rapid development of Stage 3 T1D (HR = 4.52, p = 0.014), followed by the combination of GADA and IAA (HR = 2.82, p < 0.0001) | Small sample size  Prevalence data  ⊕⊕⊕⊖ MODERATE  Predictors  ⊕⊕⊕⊖ MODERATE |
| Triolo et al.  [49] | Prospective | Youths IAb+ 246 children diagnosed with DT1  vs 453 not diagnosed with T1D  Name of the study: DPT-1  Age: median 11.4 vs 15.25 y  f/up: not reported Period: 2007 Region: USA | ICA, IAA | HLA OGTT every 6 months HbA1c | The majority of youths diagnosed with T1D through the screening were asymptomatic at onset (63.3%)  Only 8 (3.67%) presented with ketosis  35.4% had normal fasting glucose (<100 mg/dl) at the time of the diagnosis although 2-h glucose results < 200mg/dL are less frequent; they had normal HbA1C levels (6.41±1.15%)  The amount of decline in C-peptide becomes more substantial with increasing time after diagnosis  Screening with HbA1c will miss identifying many of individuals with Stage 2 ot Stage 3 T1D in this cohort |  | Only 2 IAb tested, f/up not reported  Prevalence data  ⊕⊕⊕⊖ MODERATE |
| Elding Larsson HE et al. [50] | Retrospective | 424,788 children at genetic risk, enrolled 8.677  Data on children < 2 y and < 5 y are analyzed  Name of the study: screened in TEDDY study  Comparison with children with T1D onset during the same periods in TEDDY countries (SEARCH, Swedish, Finnish, German DPV Diabetes Registers  F/up: up to 15 y of age  Period: 2004-2010  Region: USA, Finland, Sweden, Germany | GADA, IA‐2A, IAA  From 3 m of age to 15y, every 3m  In IAb+: random BG, HbA1c at each visit (3m)  For IAb+ children >3 y of age: OGTT every 6 m | Comparisons of TEDDY data on DKA vs other registers | Stage 3 T1D developed in 119 children < 5 y 40 children < 2 y 79 children <5 y  DKA rate at onset:  6/40 (15%) <2 y, lower than in comparative registries (p< 0.0001)  9/79 (11.3%) <5 y, lower vs SEARCH and German DPV (p< 0.0001) but not compared with Swediabkids (16.9%) (P = 0.45) or Finnish (18.7%) (P = 0.11) registers |  | Retrospective, bias of interpreting data with a different definition of DKA used in each study  Prevalence data  ⊕⊕⊕⊖ MODERATE |
| Ghalwash M. et al. [57] | Prospective | 6722 children at high risk of T1D  Names of the studies: DIPP, BABYDIAB, DiPiS, DAISY, DEW-IT  Age: < 2yrs  F/up: until 15 y of age (6050)  or T1D onset (n=672) Region: Europe | GAD, IA-2, IAA  One or two fixed ages | IAb and blood glucose | Optimal screening ages for two measurements were 2 and 6 y of age  Abs positivity at the beginning of each test age was highly predictive of diagnosis in the subsequent 2–5.99 y or 6–15-y intervals.  Abs usually appeared before age 6 years even in children diagnosed with T1D much later in childhood  672 developed Stage 3 T1D by the age of 15 y | Sensitivity of 82% (95% CI 79–86) and PPV of 79% (95% CI 75–80) for T1D by age 15 y | Prevalence data  ⊕⊕⊕⊖/⊕⊕⊕⊕ MODERATE  Predictors ⊕⊕⊕⊖/⊕⊕⊕⊕ MODERATE |
| Siljander HT et al. [58] | Prospective | 218 children at high genetic risk with > 1 IAb+  Name of the study: DIPP  Age: newborn  f/up: 6 y Period: from 2006  Region Finland | IAA, GADA, IA-2A, ICA  According to DIPP study  IVGTT at least  Abs obtained at least in at least  two consecutive samples, taken with a minimum of  3 m apart | IAb IVGTT (FPIR, HOMA-IR) | Progressors (n = 151, 69%) and non-progressors (n = 67) had similar distributions for baseline variables | Progressors were younger at seroconversion, had higher levels of IAb, lower FPIR and HOMA-IR, higher HOMA-IR/FPIR  A low FPIR (>24 mU/l) identifies the future cases of T1D with high accuracy (5-year progression rate >80%). Children with reasonably high insulin secretion (FPIR >59 mU/l) remain unaffected for several years | Small sample size, short f/up  Prevalence data  ⊕⊕⊕⊖ MODERATE  Predictors ⊕⊕⊕⊖ MODERATE |
| Vehik K et al. [59] | Prospective | 707 in TEDDY with genetic risk and IAb+  vs 1,190 youth from Trialnet to validate the approach  Name of the study: TEDDY  Age: 11.1 (IQR 9.0–12.8 y)  F/up: until 11.1 y or T1D onset  Period: 2004-2012 | GAD, IA-2A, IAA | In IAb+: HbA1c and IAb were measured quarterly | 235 (33.2%) developed Stage 3 T1D during f/up. No. 213 (52.9%) from the group with multiple IAbs+ and 22 from those with single IAb. | A relative increase ≥10% in HbA1c from baseline best marked the increased risk of Stage 3 T1D in TEDDY (73.6% sensitive; 88.3% specific; PPV 76%) and was as informative as OGTT 2-hPG  Predictors of risk for HbA1c change were:  age at the baseline test, (HR 0.92, overall p < 0.014) HbA1c (p < 0.0001), sex, number of IAb, rate of HbA1c increase by time of change | No HbA1c before 2009    Prevalence data  ⊕⊕⊕⊖ MODERATE  Predictors ⊕⊕⊕⊖ MODERATE |
| Xu P et al.  [60] | Prospective | 144,295 FDR  n=1073 cohort 1: 1 IAb  n=1826 cohort 2: ≥ 2 Iab  n=1444 cohort 3: with dysglycemia (at least one abnormal OGTT result)  Age: median 11, 13, 17 y  F/up: median 2 y (cohort 1), 1.6 y (cohort 2), 1.8 y (cohort 3)  Period: 2001-2005  Region: USA | GAD65, mIAA, IA-2A, ZnT8, ICA  Individuals positive for at least one Iab were followed longitudinally  for the development of additional Iab (including ZnT8 and ICA) dysglycemia, and T1D | OGTT in individuals Iab+  F/up: OGTT every 6 m | Stage 3 T1D developed in a total of 414 individuals with dysglycemia (28.7%) during further f/up  f/up time between dysglycemia and Stage 3 T1D was 1.8 years (IQR 0.8–3.6)  Overall 5-year risk if developing Stage 3 T1D was 42% | The 5-year risk of progression from single to multiple Iabs was 11% for  those individuals >16 y of age with low GADA titers, 29% for those ≤16 y of age with low GADA titers, and 45% for those individuals with high GADA titers  Progression to dysglycemia was associated with: IA-2A titers, and 2-h glucose and fasting C-peptide  Progression to Stage 3 T1D was associated with: the number of Iab+, peak C-peptide level, HbA1c level, and age | Short f/up  Prevalence data  ⊕⊕⊕⊖ MODERATE  Predictors ⊕⊕⊕⊕ HIGH |
| Redondo MJ et al.  [61] | Prospective | 354 individuals with IAb+, with at OGTT 120’ BG > 11.1 mmol/L and/or Index60 ≥ 2.00  Name of the study: Trialnet PTP  Age: median 11.2 y (1.7-46.6)  f/up: median 3.6 y Period 2004-2018  Region USA | GAD, IAA, IA-2A, ICA512 | Participants were monitored with IAb testing, HbA1c and OGTTs at 6- or 12-month intervals depending on estimated risk  OGTT, fasting c-peptide, index 60 (fasting C-peptide, 60 min C-peptide and 60 min glucose) | N=76 Glu(+): 120’ BG > 11.1 mmol/L and Index60<2.00  N=113 Index60(+): 120’ BG < 11.1 mmol/L and Index60≥2.00  N=165 both 120’ BG > 11.1 mmol/L and Index60≥2.00 | Participants in the Index60 (+) group had more typical characteristics of T1D than participants in the Glu (+) group.  Glu (+) group had a substantially higher C-peptide response than the other two groups did  Index60 (+) had a 4-year cumulative Stage 3 T1D incidence of 95% | Small sample size, short f/up  Prevalence data  ⊕⊕⊕⊖ MODERATE  Predictors ⊕⊕⊕⊖ MODERATE |
| Ismail HM et al.  [62] | Prospective | 2835 children IAb+ 298 progressors to T1D 2216 non progressors  Name of the study: TNPTP study  Age:  Progressors: median 10.5y Non progressors: 12.7 y  f/up: 3.8±2.7 y Period: 2007 Region: USA | GAD, IAA, IA-2A, ICA512 | Changes from first to last OGTTs, C-peptide every 6-12 months | 298 progressors to Stage 3 T1D (10.5%) | Progressors had a shorter interval between the first and last OGTT (1.7 years vs. 2.1 years) compared with non-progressors and they were more likely to have a Glucose Response Curve change from biphasic (two peaks) to monophasic (one peak) (75.4% vs 51% respectively, p < 0.001) to monotonic (continuous increase) during the progression to Stage 3 T1D  These GRC changes correspond to decreasing beta cell function, as the early (30-0 min) C-peptide response decreased in progressors | Short f/up  Prevalence data ⊕⊕⊕⊖ MODERATE  Predictors ⊕⊕⊕⊖ MODERATE |
| Steck AK et al. [63] | Prospective | 23 IAb+ participants with CGM data  Name of the study: DAISY  Age: Progressors: 13.9± 3.8 Non-progressors: 16.6±4.5 Period: 2011 F/up: median 2.1 y: 25.6 m (IQR 5.3 to 55.9 m)  Region: USA |  | Baseline 5- to 7-day period of blinded CGM (Dexcom SEVEN Plus System (before July 2014) or Dexcom G4 (starting 1 July 2014) | 8/23 progressed to Stage 3 T1D at a median age of 13.8 y (11.9 to 19.1) and during a median f/up of 17.7y (14.6-22) | Progressors showed at baseline increased mean BG (129 vs 104 mg/dL; p = 0.02), daytime sensor average (122 vs 106 mg/dL; p = 0.02), daytime sensor area under the curve (AUC, 470,370 vs 415,465; p = 0.047), glycemic variability (SD, 29 vs 21 mg/dL; p = 0.047). They spent 24% of time at >140 mg/dL and 12% at >160 mg/dL compared with 8% and 3% for non-progressors (both p = 0.005).  The cutoff of 18% time spent at >140 mg/dL had 75% sensitivity, 100% specificity, and a 100% PPV for Stage 3 T1D prediction after 25.5 m (median) | Sample size Short f/up  Prevalence data  ⊕⊕⊖⊖ LOW   Predictors  ⊕⊕⊕⊖ MODERATE |
| Steck AK et al. [64] | Prospective | n = 91 children IAb+ with a baseline CGM  Name of the study: ASK  Age 1-17 y (median 11.5) f/up: median 6 m Period: 2017-2019 Region USA | Baseline | HbA1c testing every 3–6 months    Optional OGTTs and blind CGM (7-10 days with Dexcom) every 6 months after the baseline monitoring visit | 16 (18%) progressed to Stage 3 T1D (median 4.5 y) | Progressors had: -significantly increased glycemic variability (SD 27 vs 16, CV 21% vs 15%, mean of daily differences 24 vs 16, and mean amplitude of glycemic excursions 43 vs 26, all p < 0.001) -21% of time spent > 140 mg/dL, 8% > 160 mg/dL vs 3 and 1% respectively for non-progressors    The risk of progression to Stage 3 T1D in 1 year was 80% in those with time >140 mg/dL of >10% vs 5% in participants with Time >140 mg/dL of <10%  Time spent > 140 of >10% is proposed as a new criterion for dysglycemia (88% sensitivity, 91% specificity and 67% PPV) | Short f/up, Limited number of OGTT (optional)  Prevalence data  ⊕⊕⊕⊖ MODERATE  Predictors  ⊕⊕⊕⊖ MODERATE |
| Ylescupidez A et al. [65] | Prospective | n=93: Stage 1 (n=58), Stage 2 (n=35)  Name of the study: Trialnet PTP  Age: median age 16.8 y  F/up: 1 y  Period: 2015–2018 Region: USA | GAD, IAA, IA-2A, ICA512 | CGM Dexcom G4 for up 7 days at baseline, 6 and 12 m    OGTT | 5/7 OGTT metrics and 29/48 CGM metrics differed between progressors and not to T1D | In multivariate analysis, OGTT-derived variables had higher discriminative ability than CGM metrics  CGM measures in multiple IAb+ individuals are predictive of T1D but less than OGTT derived variables | Short f/up. Further studies to confirm the result  Predictors  ⊕⊕⊕⊖ MODERATE |
| Desouter AK et al. [66] | Prospective | 34 FDR multiple IAb+ (No. 2 Stage 2)  Name of the study: BDR  Age: 16.6y (13.4-23.4) in IAb+  f/up: 3.5 y (2.0-7.5)  Period: 2021–2023 Region: Australia  IAb+: 16.6y (13.4-23.4) | GAD, IAA, IA-2, ZnT8 | 5d-CGM  HbA1c and OGTT  every 6m | 17 progressors to Stage 3 T1D after a median of 40m  17 non-progressors (No. 2 Stage 2 T1D) | Baseline predictions (cross-sectional):  In rapid progressors, glycemia was 4.3% of the time >140 mg/dL and 14.5% of the time >120 mg/dL, compared with with 0.0% and 1.1% in non-progressors respectively (p < 0.001) CGM metrics equaled OGTT measures. OGTT-based multivariable models remained superior  Longitudinally: repeated CGM only if associated with HbA1c were nearly as effective as OGTT in predicting stage 3 T1D | Prevalence data  ⊕⊕⊖⊖ LOW  Predictors  ⊕⊕⊕⊖  MODERATE |
| Wilson DM et al. [67] | Prospective | 105 FDR Stage 1 (n=53) with normal OGTT, Stage 2 (n=42) with abnormal OGTT, and a control group IAb negative (n=10)  Name of the study: TrialNet PTP  Age: median age 16.8 y  F/up: 1 y  Period: 2015–2018 Region: USA | GAD, IAA, IA-2A, ICA512 | CGM Dexcom G4 for up 7 days at baseline, 6 and 12 m    OGTT | No. 29/95 IAb+ (30.5%) developed T1D | Spending ≥5-8% of the time with glucose >140-160 mg/dL (p=0.02) was a good predictor of progression to Stage 3 T1D  Stage 2 participants and those who progressed to Stage 3, also exhibited higher mean daytime glucose values, spent more time > 120-140-160 mg/dL and had greater variability  CGM could aid in identification of individual, including those with a normal OGTT, who are likely to rapid progress to Stage 3 T1D | Short f/up  Predictors  ⊕⊕⊕⊖ MODERATE |
| **OTHER STUDIES WITH LOW LEVEL OF EVIDENCE, NOT INCLUDED IN THE RESULT SECTION (No. 13)** | | | | | | | |
| Sims EK et al. [10] | Prospective | 246 children Iab+  (DPT-1)  503 children Iab+ (TNPTP)  with similar baseline metabolic impairment  94 children Iab- (TNPTP)  Name of the study: DPT-1, TNPTP  Age: < 18 y  f/up: 3-4 years Period: from 2000  Region: USA | GAD, IAA, IA-2A, ZnT8 | Iab+ relatives had baseline 2-h OGTTs and then underwent bi-yearly OGTTs with glucose and C-peptide |  | Similar abnormal baseline risk,  and within 6 m progressors to Stage 3 T1D had decrease in C-peptide AUC and appreciably more metabolic impairment from baseline than non-progressors: AUC glucose, p ≤ 0.007; AUC ratio, p ≤ 0.034; Index60, p < 0.001; vector indices of change, p < 0.001  Differences in 6-month change from baseline were positively associated with greater diabetes risk, except AUC ratio  Longitudinal metabolic patterns did not differ between non progressor and IAb- | Short f/up  Prevalence data  ⊕⊕⊖⊖ LOW  Predictors ⊕⊕⊖⊖  LOW |
| Simmons et al. [11] | Cross-sectional | 478 individuals attending 39 health fairs  Age: 1-18 y F/up: - Period: 2015-2018 Region: USA | GADA, IA-2A, IAA and ZnT8A (DBS in filter paper) | Venous sample: IAb, HbA1c and BG | 9 out of 478 patients (1.9%) with positive IAb at the DBS: 8 reported single IAb, one with multiple IAbs  Strong correlation between DBS sample measurements and serum for GADA (r^2^ = 0.99, P < 0.01), IA-2A (r^2^ = 0.99, P < 0.01) and ZnT8A (r^2^ = 1.0, P < 0.01), but not for IAA Screening for T1D risk at community health fairs using DBSs on filter paper is feasible |  | Small sample size, Cross-sectional.  Prevalence data  ⊕⊕⊖⊖ LOW |
| Eising S et al. [13] | Retrospective case control | 6069 (2023 T1D, 4042 matched controls)  Age: 0-23 y Follow-up: 0-23 y Period: 1981- 2002 Region: Denmark | GAD-65, IA-2A  on DBS samples collected 5 days after birth. RBA (cut-off 31 RU/ml for GADA and 5 RU/ml for IA-2A)  HLA-DQB1 genotype | IAb, visits | The median age at Stage 3 T1D diagnosis was 8.8 years (range 1–22 years)  GAD65A and IA-2A were found in 70 (3.5%) patients vs 21 (0.5%) controls, with a HR of 7.49 (P<0.0001)  The HR decreased to 4.55 but remained significant (P<0.0003) after controlling for parental diabetes and HLA-DQB1 alleles | GAD65A and IA-2A positivity at birth are associated with an increased risk of developing Stage 3 T1D in Danish children, regardless of HLA risk genotypes and maternal diabetes | Retrospective  Only 2 IAb  Prevalence data  ⊕⊕⊖⊖  LOW  Predictors  ⊕⊕⊖⊖  LOW |
| Huber E et al. [17] | Prospective | 97 children and adolescents, general population  Name of the study: Fr1da  Age: 10y median (range 3-17)  f/up: 6 m  Period: 2015-2023  Region: Germany | IAA, GAD, IA2, ZnT8 | 10d-CGM blinded (and after 6m in 18 youths)  OGTT at T0 and after 6m | At baseline, 64 with multiple IAb+ (46 with Stage 1 and 18 with Stage 2 T1D) and 33 IAb-  11/64 developed Stage 3 T1D | Using thresholds corresponding to 100% specificity in controls, differences between controls and stage 1 and stage 2 were obtained for glucose SD > 29 mg/dL, TA140 >50%, TA160 > 14%, and TA180> 4%  Elevations in any 2 of these parameters identified 12 (67%) with Stage 2 and 9 (82%) of 11 participants who developed Stage 3 T1D within 1 year, with marked variation within groups for all parameters and poor consistency observed in the second CGM | Predictors  ⊕⊕⊖⊖ LOW |
| Xu P et al.  [43] | Prospective | 339 individuals ICA +, with low FPIR from an IVGTT and/or detection of IGT from an OGTT at baseline  Name of the study: Subcohort from the DPT-1 parenteral study  Age: 3–45 y for FDR and 3–20 y for second-degree relatives  F/up: 5 y (median of 3.7 y) or T1D onset  Period: 1994-2003  Region: USA, Canada | ICA, IAA, ICA512A (IA2), GAD | ROC curve analysis  Baseline metabolic markers from IVGTT: FG, fasting insulin, FPIR, HOMA-IR, FPIR/HOMA  Baseline metabolic markers from OGTT:  FG, 2-h glucose, peak C-peptide, AUC C-peptide | Stage 3 T1D rate: 139/339 individuals (41%) | Prognostic values for 5-y risk progression to Stage 3 T1D: Individually: IAA titer, ICA512A titer, peak C-peptide, 2-h glucose, FPIR, and FPIR/HOMA-IR (similar level of AUC for ROC ranging between 0.61 and 0.67)  The combination of 3 OGTT markers (2-h glucose, peak C-peptide, and AUC C-peptide) significantly improved the prognostic accuracy compared with any solitary index (p < 0.05) with an AUC of 0.76 (95% CI 0.70-0.81)  No variation for the prognostic accuracy by the addition of IAb titers (IAA and ICA512A) and/or IVGTT markers (FPIR and FPIR/HOMA-IR) (p = 0.46 and p = 0.66, respectively) | Small sample size Short f/up  Prevalence data  ⊕⊕⊖⊖ LOW  Predictors ⊕⊕⊖⊖ LOW |
| Åkerman L et al. [68] | Prospective | 21 high‐risk children, multiple IAb+ on ≥ 2 sampling occasions  Name of the study: Subcohort from 17.055 children from the general population screened within the ABIS study  Age: 0-12 y  F/up: 2 y  Period: 1997- 1999  Region: South‐east of Sweden | GADA, IA-2A at birth and at 1, 2.5 to 3, 5 to 6, 8, and 11 to 12 y of age   IAA from 5 to 6 y of age | HLA genotype at baseline  Visits, FG, HbA1c, C–peptide, IAb (GADA, IA2A, ZnT8A, IAA) every 6 m  OGTT yearly  PBMC | 9/21 had low risk HLA genotype 12/21 progressed to Stage 3 T1D (57%)  No difference in the occurrence of IGT and/or IFG during the f/up between the 2 groups | Children with increased HLA‐risk had higher FG at the 6 months visit (p = 0.009).  In progressors:  - increase of HbA1c over time in spite of increase of C‐peptide levels  - levels of IA2A (P = 0.058) and ZnT8A were higher than non-progressor | Sample size, short f/up  Prevalence data  ⊕⊖⊖⊖  VERY LOW  Predictors  ⊕⊕⊖⊖  LOW |
| Andersson et al. [69] | Cross-sectional | 47 children with GAD65 and at least one more IAb  Age: 4-18 y Period: 2009-2012 Region: Sweden  F/up:- | HLA GADA and at least 1 more IAb | IvGTT and OGTT were performed 8-65 d apart  FG, insulin, c-peptide, HbA1c | 20/47 had impaired glucose metabolism: 14/20 had decreased FPIR (≤ 30uU/mL insulin); 11/20 had IGT in the OGTT  Five had both IGT and FPIR ≤ 30uU/mL  Number and levels of IAb were not associated with glucose metabolism, except for an increased frequency and level of ZnT8QA was associated with impaired glucose metabolism |  | Sample size, cross-sectional  Prevalence data  ⊕⊕⊖⊖  LOW |
| Liu Y et al. [70] | Cross-sectional | 240 FDR  Age: median 15.5 ys (range 1-49 yrs)  F/up: -  Period: 2015  Region: USA | GADA, IA-2, ZnT8, IAA  Iab + ≥1 had confirmatory venous testing | None | 4/5 Iab+ had confirmatory venous testing  Capillary collection was considered uncomfortable or painful by 43% of participants  82% preferred home capillary sampling vs outpatient venipuncture  Preference for capillary sampling varied by age: 90% of those aged ≤8 yrs, 83% of those aged 9-18 yrs and 73% of those aged >18 yrs, with greater preference among younger children (p=0.01)  Self-collected capillary blood sampling offers a feasible alternative to venous sampling, with the potential to facilitate Iab screening for T1D risk |  | Cross -sectional, small sample size, no f/up  Prevalence data  ⊕⊖⊖⊖ VERY LOW |
| Martinez MM et al.  [71] | Prospective | 30 participants with a single IAb+  vs 46 participants with ≥ 2 Ab+  Name of the study: Subcohort from TEDDY, DiPiS, DIPP study, TrialNet screening  Age: 2-49.99 y  F/up: -  Period: 2015  Region: Sweden and Finland | GADA, IA‐2A, IAA, ZnT8 | OGTT for single Iab+  IvGTT for multiple Iab+  Glucose, insulin, C‐peptide and HbA1c from OGTT and IvGTT samples | Participants with a single Iab+ had:  - NGT test (with 120 minutes < 7.70 mmol/L)  - normal HbA1c values (<42 mmol/mol)  - Insulin responses on OGTT ranged between 13.0 and 143 mIU/L  - C‐peptide values between 0.74 and 4.60 nmol/L  In the multiple Iab+  - the FPIR on IvGTT was lower in those positive for ≥ 3 Ab+ (83.0 mIU/L; range 20.0‐343) vs 2 Ab+ (median 146 mIU/L; range 19.0‐545; p = 0.0330)  In conclusion. Those who had a single Iab+ appeared to have a normal beta cell function, ≥ 3 Iab+ had a lower FPIR |  | Sample size, no f/up. Different clinical laboratories for measurements of glucose, insulin, C‐peptide  Prevalence data  ⊕⊖⊖⊖ VERY LOW |
| Montaser E et al.  [72] | Prospective | 60 healthy individuals FDR with CGM data  Name of the study: TPP  Age: 23.7 +/- 10.7 Period: 2019  f/up: - Region: USA | Participants divided in 3 groups:  0 IAb (n=21)  1 IAb (n=18) ≥ 2 IAb (n=21)  Use of machine learning technology | Blinded CGM application for 7 days (n=12)  Consummation of three SLMM (standard liquid mixed meals) to replace breakfast | Among all computed glycemia metrics, only three were different across the IAb groups: percent time > 180 mg/dL (T180) weekly (p = 0.04), overnight CGM incremental AUC (P= 0.005), and T180 for 75 min post-SLMM CGM traces (p=0.004) |  | Small sample size. No f/up. No data on Time spent >140 mg/dL  Prevalence data  ⊕⊕⊖⊖  LOW |
| Steck AK et al.  [73] | Retrospective | 68 children who had a complete baseline OGTT and ≥1 confirmed IAb+  Name of the study: DAISY  Age: birth-30  Period: 1993-2004  F/up  IAb+ non progressor to T1D 6.9±3.2 y  IAb+ progressor to T1D 3.6±2.9 y  Region: US | IAA, GAD65, IA2; ZnT8  Baseline OGTT | Baseline OGTT | n=43 IAb+ non progressor to Stage 3 T1D  n=25 IAb+ progressor to Stage 3 T1D after a mean follow-up of 5.7 y and at a mean age of 12.4 y  Age at Stage 3 T1D onset or last visit:  IAb+ non progressor to T1D 17.1±4.8 y  IAb+ progressor to T1D 12.4±3.5 y | Factors that predicted progression to Stage 3 T1D:  Age at seroconversion, number of IAb+, IA-2A and ZnT8A levels, HbA1c, 1h glucose, 2h glucose, glucose AUC, glucose sum, 1h C-peptide, C-peptide AUC, C-peptide sum and index 60  On the other hand, HLA DR3/4, BMI, FDR with diabetes, levels of IAA or GADA, fasting glucose, fasting C-peptide and 2 h C-peptide did not predict progression to diabetes  DAISY IAb+ individuals who progressed to T1D had a younger age at seroconversion (5.4 ± 2.9 vs 8.1 ± 4.1 yrs respectively, p=0.005) | Retrospective  Small sample size, short f/up  Prevalence data  ⊕⊕⊖⊖  LOW  Predictors ⊕⊕⊖⊖  LOW |
| Stene LC et al. [74] | Prospective | 92 children ≥1 confirmed IAb+ and had HbA1c and random BG  Name of the study: DAISY  Age: birth-30 y  F/up: 3.4 y (mean) from onset of autoimmunity  Period: 1993-2004  Region: US | GAD65, IA-2, IAA  Timing of f/up: every 3-6 months | HbA1c and random venous glucose | 28 (30%) children developed Stage 3 T1D at a mean age of 6.5 y. Mean pre-diagnostic HbA1c was 5.1% [standard deviation (SD) =0.4%] Increase in HbA1c predicted increased risk of progression toStage 3 T1D, HR = 4.8 (95% CI 3.0–7.7) for each SD of 0.4%, independent of random BG and number of IAb.  Increase in random BG levels only marginally predicted risk of progression (HR = 1.4, 95% CI 1.02–1.8, per SD of 1.1 mmol/L) |  | Small sample size, Short f/up  Prevalence data  ⊕⊕⊖⊖ LOW |
| Warncke K et al. [75] | RCT | 1050 children at high genetic risk (RCT oral insulin vs placebo), 77 with IAb+  Name of the study: POInT  Age: 4 m – 3.6 y  F/up: 1.46 y Period: 2017 Region: German, Poland, UK, Belgium, Sweden | GAD, IA2, IAA, ZnT8 | Pre-prandial and post-prandial BG and IAb at 2, 4, 8, 18m and then every 6 m until 7.5 y of age | BG values were highest in infancy and decreased to a nadir at 12 to 18 m of age, influenced by sex, BMI, and genetic factors, including the T1D susceptible INS gene  BG values increased until the final measurement at 3.6 y of age | Increased BG concentrations were observed in children who developed IAb+, first in 30-minute postprandial blood glucose (occurred at around 2 m prior to IAb seroconversion), and subsequent rises in pre-prandial values shortly after seroconversion | Short f/up, Small IAb+, sample size  Prevalence data  ⊕⊕⊖⊖ LOW  Predictors ⊕⊕⊖⊖ LOW |

**Abbreviations:** Year/s (y); month (m); type 1 diabetes (T1D); first degree relative (FDR); general population (GP); diabetic ketoacidosis (DKA); islet autoantibody (IAb); human leukocyte antigen (HLA); follow-up (f/up); height (HT); weight (WT); dried blood spot (DBS); radiobinding assay (RBA); GADA (glutamic acid decarboxylase antibodies); GAD65 antibody (GADA); islet cell antibodies (ICA); antibodies to ICA-512 (ICA512A); insulin autoantibodies (IAA); insulinoma antigen-2 autoantibodies (IA-2A), zinc transporter 8 antibody (ZNT8A); M (maintainers); R (reverters); glycated hemoglobin (HbA1c); oral glucose tolerance test (OGTT); continuous glucose monitoring (CGM); diabetic ketoacidosis (DKA); fasting glucose (FG); blood glucose (BG); hazard ratio (HR); positive predictive value (PPV); impaired fasting glucose (IFG); impaired glucose tolerance (IGT); intravenous glucose tolerance test (IVGTT); homeostasis model assessment of insulin resistance (HOMA-IR); area under the curve (AUC); receiver operating characteristic (ROC); first-phase insulin response (FPIR); All Babies in Southeast Sweden (ABIS); Autoimmunitiy Screening for Kids program (ASK); Diabetes Prevention Trial–Type 1 (DPT-1); Diabetes Prediction and Prevention Study (DIPP); Diabetes Prediction in SKane Study (DiPiS); TrialNet’s Pathway to Prevention (PTP); The Environmental Determinants of Diabetes in the Young (TEDDY); Diabetes Prevention Trial Risk Score (DPTRS); Type 1 Diabetes Intelligence (T1DI)
