# Supplementary material for: Impact of screening programmes for type 1 diabetes in youth: A systematic review and meta‐analysis
Source: Diabet Med. 2026 Jan 31;43(5):e70236. doi: 10.1111/dme.70236 (PMC13074144; doi:10.1111/dme.70236)
Supplement: Supplementary file 3 — Table S3: Literature analysis after PICOS selection: summary of the studies and evidence grading for each study that reported prevalence data and predictors of psychological outcomes. [file DME-43-e70236-s002.docx]

**Supplemental Table S3:** Literature analysis after PICOS selection: summary of the studies and evidence grading for each study that reported prevalence data and predictors of psychological outcomes.
Data are expressed as mean±SD, unless otherwise stated. Quality of evidence: High ⊕⊕⊕⊕, Moderate ⊕⊕⊕⊖, Low ⊕⊕⊖⊖, Very Low ⊕⊖⊖⊖.

| **Reference** | **Study design** | **Sample, age and comparator** | **Screening (Abs, age)** | **Modality of f/up participation in trials** | **Prevalence data** | **Predictors** | **Study limitations,  risk of bias  and level of evidence for prevalence data and for predictors** |
| --- | --- | --- | --- | --- | --- | --- | --- |
| **General Population** | | | | | | | |
| Ziegler  AG et al. [16] | Prospective | 90,632 healthy children screened and 264 parents from DiMelli cohort for psychological outcomes    Age: 1.75 to 5.99  f/up: 2.4 (1.0-3.2) y  Period: 2015-2019  Region: Bavaria (Germany) | GADAs, IA-2A, ZnT8A (capillary) | Interval 2-6m  PHQ-9 for Parental psychological stress | Psychological stress scores significantly increased at the time of metabolic staging in mothers of children with IAb+ vs mothers of children IAb- (p = 0.002) but declined after 12 m of f/up (p < 0 .001) |  | Short f/up  Prevalence data  ⊕⊕⊕⊖ MODERATE |
| Melin J et al. [53] | Prospective | Parents of 2088 children screened at birth and followed for at least 5 y were included  F/up: 5 y  Up to 4 y: 1986 IAb-, 79 had 1 IAb, 23 had multiple IAb+  Name of the study: DiPiS  Period: September 2000 -August 2004  Region: Sweden | GAD65A, IA-2A, IAA, Znt8A | Parental anxiety: STAI with 6-items, annually from 2 to 15 y  Parental frequency of worry assessed with one semi-structured question  Risk perception assessed with one semi-structured question  No f/up of IAb-  F/up of IAb+: annually from 2 y to 15 y  Children with one IAb+: contact by phone for the risk information; no more contact if stable IAb+  Children with multiple IAb or progressing from one to multiple IAb+: f/up every 3 m with HbA1c, FBG and annually OGTT and IAb  After 3 y, they were asked if they wanted to know the genetic risk | No association between participation in the DiPis and anxiety after 5 y in 67.1% of mothers and fathers  In 7.1% of families, both parents were anxious that their child would develop Stage 3 T1D in the future  Frequency of worry was associated with parental anxiety (p < 0.001 for both mothers and fathers) | Anxiety was higher in: - mothers of children positive for IAb (p<0.001) and those perceiving their child had higher risk for Stage 3 T1D (p<0.001).  - having an FDR with Stage 3 T1D (p<0.001 in mothers and 0.004 in fathers)  Anxiety was less in highly educated parents (p<0.001) | Short f/up Absence of control group  Prevalence data  ⊕⊕⊕⊖  MODERATE  Predictors  ⊕⊕⊕⊖  MODERATE |
| Smith LB et al. [56] | Prospective | 8676 patients HLA+, including general population children and FDR with T1D  Name of the study: TEDDY  Age: 0-15 y F/up: up to 15y Period: 2004-2015 Region: USA/Europe | GADA, IA-2A and IAA | Blood test  Psychological test  F/up  every 3 m during the first 4 y of life, every 6 m thereafter up to 15 y or the diagnosis of Stage 3 T1D | Among FDRs, 7–10% reported watching for symptoms and 7–9% reported monitoring the child's BG  After IA+ notification, all monitoring behaviors significantly increased in general population parents; only BG monitoring increased in FDR parents and these behaviors continued for up to 4 y | FDR status, accurate diabetes risk perception, and anxiety were associated with BG monitoring in IA+ and IA– cohorts  > Monitoring behaviors in mothers than in fathers, in older children, first born children, non-ethnic minority children, and FDR families compared to general population families | Data self-reported Absence of control group  Prevalence data ⊕⊕⊕⊖  MODERATE  Predictors ⊕⊕⊕⊖  MODERATE |
| **High-risk /FDR** | | | | | | | |
| Goldstein E et al. [51] | Prospective | 664 parents of newborns (457 HLA+; n =188 ≥ 2 IAb+, 19 progressed to T1D)  vs 843 matched parents, whose child were HLA+ and IAb-  RCT intranasal insulin vs placebo (1997-2007)  Name of the study: DIPP study  Age: newborn  f/up - The time from notification of IAb+ to T1D ranged from 0.5 to 6.7 y (mean 2.9)  Period: 1997-2005  Region: Finland | GAD, IA2, IAA | Parental anxiety assessed with PSI with 11 items | Stress indexes were similar in parents of IAb+ children and control  Fathers experienced less stress than mothers  Transient IAb+ or the presence of multiple permanent IAb in the child did not alter parental stress level  Parental stress was similar whether or not the child participated in the prevention trial  Parents whose child had developed Stage 3 T1D showed higher stress than controls. Parental stress decreased with duration of the f/up (r=0.142, p = 0.001) | Single parents, urban environment, unemployment and chronic illness in the family were associated with higher stress. As well as with maternal age (r=-0.115, P=0.039) but not with paternal age | Small sample size, F/up not reported  Prevalence data  ⊕⊕⊕⊖  MODERATE  Predictors  ⊕⊕⊕⊖  MODERATE |
| Johnson SB et al. [52] | Prospective | Parents of 6799 children with genetic risk for T1D were divided into 3 cohorts:  - 5985 children IAb-, up to 6 y age  - 814 children IAb+ and parents had completed SAI before and after the IA+  - 718 children with IA+ results who were followed for 1-4 y after the initial IA+  Name of the study: TEDDY  Age: screening before 4.5 m of age  f/up: 1-6 y  Period: 2007-2017  Region: Finland, Germany, Sweden, US  Study comparator: TEDDY | IAA, GAD, islet antigen 2 (IAA), ZnT8 measured with RBA and ECL | Parental anxiety: SAI short-form with 6-items, given at 3, 6 and 15 m and annually thereafter  Parent risk perception: assessed with one semi-structured question  Every 3 m for 4 y and every 6 m thereafter | At the first visit SAI score > 40 in 47% of mothers and 34 % of fathers. | Positively associated factors:  - FDR affected by Stage 3 T1D vs general population  Factors predicting post-IA+ SAI scores  - Parent pre-IA+ SAI score  - Risk perception accuracy (p < 0.001).  Factors associated to higher SAI scores during f/up: - The first IA+ test result (p< 0.0001)  - Two or more IA+ test (p< 0.001)  - Child being an ethnic minority (mothers only, p = 0.004) or living in the U.S. (p < 0.05)  - accurate parent perceptions of the child’s Stage 1 T1D  - Stage 3 T1D risk (p < 0.0001)  The longer was the f/up period, the lower the SAI score (p < 0.05)  Higher maternal anxiety was associated with single persistent (p = 0.008) and multiple persistent (p < 0.0001) IAb+  Higher paternal anxiety was strongly associated with multiple persistent IAb+ (p < 0.0001) | Short f/up  Prevalence data  ⊕⊕⊕⊖ MODERATE  Predictors ⊕⊕⊕⊖ MODERATE |
| O’Donnell HK et al. [54] | Prospective | 319 children 1-17 y at risk for T1D  280 (88%) with IAb+ and their caregivers were enrolled (75% with single IAb and 25% with multiple IAbs)  Name of the study: ASK program (vs TEDDY)  Age of children: 5.6-14.7  f/up: median 6.1 months  (IQR 5.3-8.5 months)  Period: 2020  Region: Denver | IAA, GAD, IA-2, ZnT8 measured with RBA and ECL | Test: HbA1c, BG, IAb  HT, WT  For multiple IAbs or a single IA by 2 methods:  CGM, BG check 2 h after the largest meal per month and daily when ill.  OGTT every 6 months  Parental anxiety: SAI with 6-items  Parent risk perception: assessed with one semi-structured question  F/up: every 3-12 m  a) if multiple IAb or a single IAb by 2 methods: every 3-6 months  b) if single IA by 1 method: every 6-12 months | At the first visit, parental SAI score > 40 in 74.4%: high anxiety about their child’s Stage 3 T1D risk  After the first 2 monitoring visits (n=109), SAI score decreased (p = 0.03), but the sample was still highly anxious | Associated factors:  - decrease parent educational attainment (high school diploma or less), Hispanic white (β = 4.9; p = 0.08), anxiety (β= 3.7; p = 0.02)  - FDR with Stage 3 T1D (β= 4.8; p = 0.01), multiple IAb (β= 2.85; p < 0.01)  Multiple regression analysis: the only predictor of change in anxiety was the initial anxiety score | Small sample size  Short f/up  Absence of control group  Prevalence data  ⊕⊕⊕⊖  MODERATE  Predictors  ⊕⊕⊕⊖  MODERATE |
| Roth R et al. [55] | Prospective | 7612 mothers of TEDDY who completed the 6 m of age questionnaire; among them, 826 had FDR  Name of the study: TEDDY  Age: enrolled before 3 months of age  f/up: 6 m (30th June 2011)  Period: 2004-2010  Region: Finland, Sweden, Germany, United States | IAA, GAD, IA-2 | At the 6 months visit, to mothers:  Parental anxiety: SAI short-form with 6 items  Negative life events scale (LE)  Maternal postnatal depression: EPDS  Parent risk perception: assessed with one semi-structured question | EPDS scores were in the normal range with an overall post-depression prevalence rate of 9% (the prevalence worldwide is estimated at 10-15%)  Country differences emerged | Highest SAI scores were dependent from:  - country (German β 1.56 and US β 0.00 mothers were considered more anxious, p<0.001);  - child ethnic minority (β 0.00, p < 0.001);  - FDR status (β 2.91, p < 0.001) ;  - maternal age (β -0.07, p = 0.006);  - education (basic primary β 0.00, trade school/college β -1.39, higher education -1.79, p < 0.001);  - maternal alcohol consumption (β -0.61, p = 0.012);  - smoking during pregnancy (β 0.76, p = 0.048)  - loss or loss threatening (β 0.77, p = 0.008)  - interpersonal negative life event (β 1.65, p < 0.001).  - postnatal depression  LE - particularly interpersonal LE - had a strong association to maternal anxiety about the baby's risk of Stage 3 T1D  Country differences emerged | Short f/up  Absence of control group  Prevalence data  ⊕⊕⊕⊖ MODERATE  Predictors  ⊕⊕⊕⊖ MODERATE |

**Abbreviations:** Year/s (y); month (m); type 1 diabetes (T1D); first degree relative (FDR); general population (GP); diabetic ketoacidosis (DKA); islet autoantibody (IAb); human leukocyte antigen (HLA); follow-up (f/up); height (HT); weight (WT); radiobinding assays (RBA); GADA (glutamic acid decarboxylase antibodies); GAD65 antibody (GADA); islet cell antibodies (ICA); antibodies to ICA-512 (ICA512A); insulin autoantibodies (IAA); insulinoma antigen-2 autoantibodies (IA-2A); zinc transporter 8 antibody (ZNT8A); Autoimmunity Screening for Kids program (ASK); Diabetes Prediction and Prevention Study (DIPP); Diabetes Prediction in SKane Study (DiPiS); The Environmental Determinants of Diabetes in the Young (TEDDY); Patient Health Questionnaire-9 (PHQ-9); State Anxiety Inventory (SAI); Parenting Stress Index (PSI); Edinburgh Postnatal Depression Scale (EPDS); quality-adjusted life-year (QALY)
