# Supplementary material for: Impact of screening programmes for type 1 diabetes in youth: A systematic review and meta‐analysis
Source: Diabet Med. 2026 Jan 31;43(5):e70236. doi: 10.1111/dme.70236 (PMC13074144; doi:10.1111/dme.70236)
Supplement: Supplementary file 4 — Appendix S1: Supporting Information. [file DME-43-e70236-s004.docx]

**Supplemental Methods**

**Search terms**

*autoantibodies OR antibodies AND screening AND population OR “general population” OR healthy AND diabetes or T1D, and the following MeSH terms were used: ((("Diabetes Mellitus, Type 1/prevention and control"[Mesh]) AND "Autoantibodies"[Mesh]) OR (("Diabetes Mellitus, Type 1"[Mesh]) AND "Autoantibodies"[Mesh] AND screening)) OR (("Diabetes Mellitus, Type 1"[Mesh]) AND "Autoantibodies"[Mesh] AND risk).*

We also screened the reference list of eligible studies to ensure all relevant studies are included. We used the “free-text search” technique for psychological outcomes to improve the performance of our search, and more documents that matched the search criteria, not selected by the electronic databases, were included.

**Inclusion and exclusion criteria**

Inclusion criteria were: i) study population: healthy children and adolescents (aged birth–18 years); ii) study type: observational studies (cohort, case-control, cross-sectional studies), exploratory studies, a mix of qualitative and quantitative studies; iii) review articles were excluded, but their reference lists were screened to identify potentially eligible studies; iv) only published full papers were included, and abstracts alone were not included; v) data on the type of screening, population age, modality and timing of follow-up, the incidence of T1D, frequency of DKA, psychological items, metabolic control, complications; vi) publication date: last 15 years (2006-2025).

Exclusion criteria were: i) population: data available only for adults ≥18 years or individuals at diabetes onset; ii) non-human animal data; iii) case reports (due to their high potential for bias—both in terms of publication and their focus on rare or unusual cases—as well as their limited generalizability); studies with <100 patients who have undergone screening; iv) full paper not available; v) study not yet published; vi) outcome: studies on screening with only genetic testing; studies that reported only the performance (sensitivity) of islet antibody kits (assay) without exploring any clinical outcome; vii) languages other than English were not an *a priori* exclusion criterion.

**Data extraction and management**

Using the search strategy, two independent investigators (RF and EMO) screened the titles and abstracts of the identified studies for inclusion. Any discrepancies were resolved by consensus or consultation with a third investigator (MM). After the abstract selection, eight investigators conducted the full text review.

The following characteristics were evaluated for each study in full papers: (i) reference details: authorship(s); published or unpublished; year of publication; period in which the study was conducted; (ii) study characteristics: study design, follow-up duration, region; (iii) population characteristics: number of participants who underwent the screening, age and demographic data; comparator characteristics; (iv) methodology: modality of screening, follow up modality; (v) main results: frequency of diabetic ketoacidosis, morbidity, mortality; psychological outcomes, educational support; authorized drugs for trials; % of progression to symptomatic T1D; frequency of pre-symptomatic diabetes; HbA1c, blood glucose, C-peptide values at the diagnosis. Data extraction was completed in duplicate.

After duplicates were removed, 1666 studies were identified through the literature review. After reviewing of titles and abstracts, 1544 records were excluded for the following reasons: 803 studies involved populations not relevant for the review; 511 reported outcomes unrelated to our focus; 67 were review articles; 1 was a case report; 88 analyzed only a single autoantibody; 50 were unavailable as full-text papers (mostly abstracts presented at conventions); and 24 studies had fewer than 100 screened youths.

A total of 122 full-text manuscripts were assessed for eligibility. After full-text examination, 56 studies were excluded: 32 not reporting outcomes of interest (impact of the screening); three for the population evaluated; eight for the population's age; seven review articles; four were letters, editorials or commentaries; two for number of participants.

Sixty-six studies were finally included in this systematic review.

**Risk of bias (quality) assessment according to the GRADE approach**Two authors (EM and RF) independently assessed the certainty of the evidence for each outcome, MM and EMA resolved discrepancies. The two authors (EM and RF) used ROBINS-I (“Risk Of Bias In Non-Randomized Studies - of Interventions”) as a tool to ensure consistency and accuracy. In cases of risk of bias in the study design, imprecision of estimates, inconsistency across studies, indirectness of the evidence, and publication bias, the recommended option of decreasing the level of certainty by one or two levels according to the GRADE guidelines was applied.^1^ Using the ROBINS-I tool, the GRADE framework assigns an initial certainty rating of high for outcome data from RCTs and low for observational studies. Certainty can be downgraded based on five domains: limitations in study design and execution (risk of bias), inconsistency (heterogeneity), indirectness (PICO and applicability), imprecision, and publication bias. Conversely, three factors can increase certainty: a large magnitude of effect, opposing plausible residual bias or confounding, and a dose-response gradient. We have reported the cut-off values (median values) used to downgrade the level of certainty by one or two levels.

The GRADE approach results in an assessment of the certainty of a body of evidence and allocation to one of four grades: high, moderate, low, or very low.

| High | Further research is very unlikely to change confidence in the estimate of the effect |
| --- | --- |
| Moderate | Further research is likely to have an important impact on confidence in the estimate of the effect and may change the estimate |
| Low | Further research is very likely to have an important impact |
| Very low | Any estimate of effect is very uncertain |

***Reasons to decrease the level of certainty by one or two levels:***

- sample size (median values as cut-off): studies including less than 564 participants with positive anti-islet antibodies (IAb+), except for IVGTT studies (less than 268 individuals) and studies with CGM (less than 91 individuals);

- possible bias due to the study design: cross-sectional, follow-up of only a few years (< 7 years = median value), only 2 IAbs tested, different assay or cut-off used in multicenter studies, different definitions of DKA used in each study, % drop-out;

- imprecision of estimates or inconsistent results between different cohorts included in the multicenter studies.

Some studies received an intermediate level of evidence (for example between moderate and high) if several outcomes were analyzed and each received a different grading.

*Data Synthesis*

Starting from Tables that reported the details of each study (Supplementary Tables), relevant information reported in the ones with moderate-high quality level of evidence, were categorized in separated “tables of evidence”, one for each outcome (Table 2 and 3).
Data synthesis was then reported in the Results section in the form of a narrative summary and as a meta-analysis where possible. Evidence statements were drawn whether evidence was available.

**Meta-analysis**

Because of the heterogeneity of study populations (ages, region, period, T1D risk) and study design (IAb assay, number of IAbs, follow-up duration and modality, absence of data from the unscreened population), it was only possible to combine the results in a meta-analysis for the outcome of the frequency of DKA in IAb+ individuals at Stage 3 T1D onset.

A meta-analysis was conducted on the impact of the screening program on the frequency of DKA at the onset of Stage 3 T1D. The analysis included studies that compared the DKA frequency in the screened and non-screened populations. In cases where data from the non-screened population were unavailable, we reported frequencies derived from the literature in populations of similar age, T1D risk, area, and period. The analysis was carried out using the risk difference as an outcome measure. A random-effects model was fit to the data. The amount of heterogeneity (i.e., tau²), was estimated using the restricted maximum-likelihood estimator.^2^ In addition to the estimate of tau², the Q-test for heterogeneity and the I² statistic are reported.^3^ In cases were heterogeneity is detected (i.e., tau² > 0, regardless of the results of the Q-test), a prediction interval for the true outcomes is also provided. Studentized residuals and Cook's distances were used to examine whether studies may be outliers and/or influential in the context of the model. Studies with a studentized residual larger than the 100 x (1 - 0.05/(2 X k))th percentile of a standard normal distribution were considered potential outliers (i.e., using a Bonferroni correction with two-sided alpha = 0.05 for *k* studies included in the meta-analysis). Studies with a Cook's distance larger than the median plus six times the interquartile range of the Cook's distances were considered to be influential. The rank correlation test and the regression test, using the standard error of the observed outcomes as predictor, were used to check for funnel plot asymmetry. A total of k=10 studies were included in the analysis.

**REFERENCES**

1. Schünemann HJ, Cuello C, Akl EA et al. How ROBINS-I and other tools to assess risk of bias in nonrandomized studies should be used to rate the certainty of a body of evidence. *J Clin Epidemiol*. 2019;111:105-114. doi: 10.1016/j.jclinepi.2018.01.012.
2. Viechtbauer W. Bias and Efficiency of Meta-Analytic Variance Estimators in the Random-Effects Model. *Journal of Educational and Behavioral Statistics*. 2005;30(3), 261-293. https://doi.org/10.3102/10769986030003261
3. Cochran WG. The Combination of Estimates from Different Experiments. *Biometrics*. 1954;10: 101–129. https://doi.org/10.2307/3001666
