# Supplementary material for: Impact of screening programmes for type 1 diabetes in youth: A systematic review and meta‐analysis
Source: Diabet Med. 2026 Jan 31;43(5):e70236. doi: 10.1111/dme.70236 (PMC13074144; doi:10.1111/dme.70236)
Supplement: Supplementary file 5 — Appendix S2: Supporting Information. [file DME-43-e70236-s001.docx]

# Supplemental Results

**Frequency of reversion from IAb+ to IAb- status**

The frequency of reversion from IAb+ to IAb- status was reported in only three studies and was relatively frequent for GAD65 (19-33%), insulin IAb (29%), and IA-2A (57%)12,42 but much rarer (4.1%) in IAb+ individuals in another cohort followed-up for 5.2 years.40 Reversion was primarily restricted to children with single IAb positivity (24%), occurred within two years of seroconversion, and was associated with the HLA genotype (DQ2 vs DQ8), decreasing IAb titers, and older age.42 In contrast, it was rare in children who developed multiple IAb+ (<1%).2 Among children in whom a single IAb+ reverted, 19-34% seroconverted back to positive40,42.

# Diagnosis of Stage 3 T1D by screening and progression during follow-up

GADA and IA-2A positivity at birth were associated with an increased risk of developing Stage 3 T1D (median age at Stage 3 T1D diagnosis: 8.8 [range 1-22] years), regardless of HLA risk genotypes and maternal diabetes.10 At 10 years of follow-up, the highest risk was detected for IA- 2+ vs GADA+ children (p=0.048)18.

The ten-year risk of progression to Stage 3 T1D was 27-69.7% in people testing positive for multiple IAbs, 14.5-26% in people testing positive for one IAb, and 0.4% in IAb- individuals24,39,44. The IAb+ number predicted progression to Stage 3 T1D: four positive IAbs vs one IAb had a hazard ratio (HR) of 33.145, while the presence of two IAbs had an HR of 1.85 (p=0.04)17. The positive predictive value (PPV) of multiple IAb positivity was higher than HLA-DQB1+ (PPV 61.1% vs 23.7%)18,45. In another study, the 15-year risk of progression to stage 3 T1D varied from 18% to 88%, based on the stringency of IAb+ definition22.

# Mortality, morbidity, hospitalization duration, HbA1c, blood glucose, C-peptide, and insulin requirements

No study reported data on morbidity or mortality related to DKA at Stage 3 T1D onset. Early diagnosis and reduced DKA rates led to a shorter hospitalization period at Stage 3 T1D onset in screened (11.4 days, 8.9-13.8) vs. non-screened individuals (14.9 days, 14.4-15.3. p=0.005)47.

Screening programs were associated with lower HbA1c28,32 and BG levels at Stage 3 T1D onset14,32 and with higher C-peptide levels and reduced insulin requirements28,32. However, this significant benefit on HbA1c, BG, C-peptide, and insulin regimen was not sustained during the first five years following diagnosis47.

# Timings

## Timing of screening

Only one study recommended annual screening starting in early childhood and continuing through early adolescence31. By contrast, the other studies proposed IAb screening at specific landmark ages: at 2-3 years, shortly after the peak incidence of IAb; at 6 years, around the time of school entry; and in early adolescence, after which the risk of developing IAb significantly decreases21,26,33.

The five-year risk of developing any or multiple IAb+ by screening at landmarked ages was reported only for the high-risk/FDR population, and it decreased with age. It ranged between 2% and 8% for any IAb+ in individuals aged <2 years21,26,33. At this age, one study reported a frequency of 6.3% for any IAb+ and of 4.3% for multiple IAb+ at 7.5 months of age21. Around 6 years, the frequency of IAb+ was 3.2% for any IAb, 1.1% for multiple IAbs21, and 0.7-0.9% at 10-12.5 years26,30.

## Timing of follow-up

The timing and methods of follow-up of the screening programs varied. Some authors recommended monitoring stage 1 (IAb+) individuals with blood tests every six months and stage 2 (with abnormal BG) individuals every three months14. Other protocols considered age at enrolment

<5 years as a factor risk for developing T1D (HR 3.21 within five years of follow-up)45.

# Modality of follow-up, metabolic monitoring, and prediction of T1D onset

## IAb positivity: type, level, single/multiple, persistence

IAA was the earliest IAb seen in children with a single IAb, with a sharp peak at age nine months24,33 and a decline over the following five years27,47. High IAA levels were seen in predominantly multiple IAbs and at ages <3 years47. GADA only increased until the second year and remained relatively constant27,50.

## OGTT

A few studies reported baseline data on OGTT in people with IAb+. Although some studies did not evaluate the relationship between OGTT and progression to Stage 3 T1D14,39,40, another study suggested that OGTT results predict Stage 3 T1D onset61. When OGTT was included in the follow- up of IAb+, the test was performed every 6 to 12 months for T1D diagnosis and/or for T1D prediction (**Supplemental table S2**)10,28,37,62. In one study, OGTT was performed every two years48. ***IVGTT***

IVGTT data of IAb+ youths were reported in five studies45,34,35,58 IVGTT improved the prognostic accuracy of 5-year progression to T1D; in particular, lower first-phase insulin response (FPIR) (<10th percentile) identified youths at T1D risk with high accuracy (five-year progression rate

>80%, HR 2.94)45,38,58, and a greater decline in FPIR was seen between 1.5 and 0.5 years before diagnosis35. FPIR and higher HOMA-IR/FPIR were of prognostic value for five-year risk T1D progression58.

## CGM

CGM data in IAb+ were evaluated in seven studies17,63-67,72, five of moderate-to-high quality (**Table 2**)63-67. Duration of blinded CGM was five to ten days; data only at baseline were considered in one study72 and every three to six months in the others17,63-67. In three studies, the accuracy of CGM metrics to predict Stage 3 T1D was compared to OGTT results65-67.
